# Pore-Forming Toxins Induce Macrophage Necroptosis during Acute Bacterial Pneumonia

**DOI:** 10.1371/journal.ppat.1005337

**Published:** 2015-12-11

**Authors:** Norberto González-Juarbe, Ryan Paul Gilley, Cecilia Anahí Hinojosa, Kelley Margaret Bradley, Akinobu Kamei, Geli Gao, Peter Herman Dube, Molly Ann Bergman, Carlos Javier Orihuela

**Affiliations:** 1 Department of Microbiology, The University of Alabama at Birmingham, Birmingham, Alabama, United States of America; 2 Department of Microbiology and Immunology, The University of Texas Health Science Center at San Antonio, San Antonio, Texas, United States of America; 3 Department of Infectious Diseases, St. Jude Children’s Research Hospital, Memphis, Tennessee, United States of America; University of Tubingen, GERMANY

## Abstract

Necroptosis is a highly pro-inflammatory mode of cell death regulated by RIP (or RIPK)1 and RIP3 kinases and mediated by the effector MLKL. We report that diverse bacterial pathogens that produce a pore-forming toxin (PFT) induce necroptosis of macrophages and this can be blocked for protection against *Serratia marcescens* hemorrhagic pneumonia. Following challenge with *S*. *marcescens*, *Staphylococcus aureus*, *Streptococcus pneumoniae*, *Listeria monocytogenes*, uropathogenic *Escherichia coli* (UPEC), and purified recombinant pneumolysin, macrophages pretreated with inhibitors of RIP1, RIP3, and MLKL were protected against death. Alveolar macrophages in MLKL KO mice were also protected during *S*. *marcescens* pneumonia. Inhibition of caspases had no impact on macrophage death and caspase-1 and -3/7 were determined to be inactive following challenge despite the detection of IL-1β in supernatants. Bone marrow-derived macrophages from RIP3 KO, but not caspase-1/11 KO or caspase-3 KO mice, were resistant to PFT-induced death. We explored the mechanisms for PFT-induced necroptosis and determined that loss of ion homeostasis at the plasma membrane, mitochondrial damage, ATP depletion, and the generation of reactive oxygen species were together responsible. Treatment of mice with necrostatin-5, an inhibitor of RIP1; GW806742X, an inhibitor of MLKL; and necrostatin-5 along with co-enzyme Q_10_ (N5/C_10_)_,_ which enhances ATP production; reduced the severity of *S*. *marcescens* pneumonia in a mouse intratracheal challenge model. N5/C_10_ protected alveolar macrophages, reduced bacterial burden, and lessened hemorrhage in the lungs. We conclude that necroptosis is the major cell death pathway evoked by PFTs in macrophages and the necroptosis pathway can be targeted for disease intervention.

## Author Summary

Necroptosis is a pro-inflammatory mode of programmed cell death that is marked by the intentional disruption of host membranes and the release of pro-inflammatory cytosolic components into the milieu. Until just recently necroptosis was not appreciated to play a role during infectious disease. Herein, we demonstrate that alveolar macrophages exposed to the nosocomial pathogen *Serratia marcescens* undergo necroptosis and this leads to enhanced disease severity. We subsequently demonstrate that necroptosis is the principle mode of cell death experienced by macrophages following their exposure to bacteria that produce pore-forming toxins (PFTs). We dissect the molecular mechanisms by which PFTs induce necroptosis and demonstrate that loss of ion homeostasis at the cell membrane and mitochondrial damage result in ATP depletion and ROS generation that together are responsible. Finally, we demonstrate that inhibition of necroptosis by various means is protective against hemorrhagic pneumonia caused by *S*. *marcescens*.

## Introduction

Necroptosis is a highly pro-inflammatory mode of cell death that is critical for immune activation following injury. Regulated by receptor-interacting serine-threonine kinases (RIP or RIPK)1 and RIP3, necroptosis involves the purposeful disruption of eukaryotic cell membranes. During necroptosis, RIP1 binds to RIP3 forming the necroptosome [1]. The necroptosome phosphorylates the mixed lineage kinase domain-like protein (MLKL), which subsequently integrates into the plasma and mitochondrial membranes leading to their disruption and the release of alarmins [2]. Unlike trauma-induced necrosis that does not involve cell signaling, necroptosis can be blocked by the inhibition of RIP1, RIP3, or MLKL [3, 4]. Necroptosis is distinct from apoptosis that is caspase-dependent and immunologically quiescent [5]. In fact, general caspase inhibitors promote cell necroptosis following receipt of a death signal such as tumor necrosis factor (TNF)α [6]. Most recently, Kitur et al. showed that necroptosis is linked to activation of the inflammasome. Inhibition of MLKL blocked caspase-1 activation and Interleukin (IL)-1β production following *Staphylococcus aureus* infection. Blocking of necroptosis protected alveolar macrophages (AMs) during Staphylococcal pneumonia and lessened disease severity in mice. Kitur et al. concluded that necroptosis was detrimental to the host during infection [7]. Importantly, the specific signals and mechanisms involved in the activation of RIP1/3 at the cellular level remained unclear.

Pore-forming toxins (PFTs) are a major class of conserved virulence determinants with an almost universal presence in pathogenic bacteria. Bacterial pathogens employ PFTs to alter the host environment and survive *in vivo* [8–10]. PFTs integrate into eukaryotic cell membranes and can induce death in distinct manners [10]. At high exposure levels, PFTs cause rapid lytic death due to the uncontrolled influx of water across the cell membrane through toxin-formed pores [11, 12]. At lower concentrations, PFTs activate cell death programs. For example, the *S*. *aureus* toxin Hla triggered necroptosis of macrophages [7]. Pneumolysin, the cholesterol-dependent cytolysin produced by *Streptococcus pneumoniae*, has been shown to damage the mitochondria of neurons and initiate caspase-independent apoptosis through the release of apoptosis inducing factor [13]. PFTs also activate the NLRP3-inflammasome, which in combination with nuclear factor kappa B (NFκB) activation, leads to the production and secretion of IL-1β and in some instances pyroptosis [14]. At sublethal concentrations PFTs also alter normal cell function. PFTs have been documented to disrupt ciliary beating on bronchial epithelial cells, inhibit macrophage phagocytosis, lead to escape from the phagolysosome, and trigger loss of tight-junction barrier integrity in the airway and gut. Thus, PFTs facilitate survival of the bacteria within an infected host and are often responsible for associated pathologies [9].

*Serratia marcescens* is a Gram-negative nosocomial pathogen that secretes a unique PFT called ShlA. *S*. *marcescens* causes a broad spectrum of infectious disease, including hemorrhagic pneumonia, and is an increasingly important cause of hospital- and community-acquired infections [15–17]. Importantly, some clinical isolates of *S*. *marcescens* have been reported to be Carbapenem-resistant [17]. Recently we have shown that during *S*. *marcescens* pneumonia, ShlA specifically depleted AMs [18]. Yet the reason for their clearance was undetermined. Herein, we demonstrate that necroptosis is the responsible mechanism for macrophage death following their exposure to ShlA. We demonstrate that necroptosis is the common response by macrophages to diverse bacterial pathogens that produce PFTs. We detail the specific cell signals induced by PFT intoxication that trigger necroptosis and show that the necroptosis pathway can be blocked at various steps for therapeutic intervention during hemorrhagic *S*. *marcescens* pneumonia.

## Results

### ShlA kills macrophages

**Fig 1:**
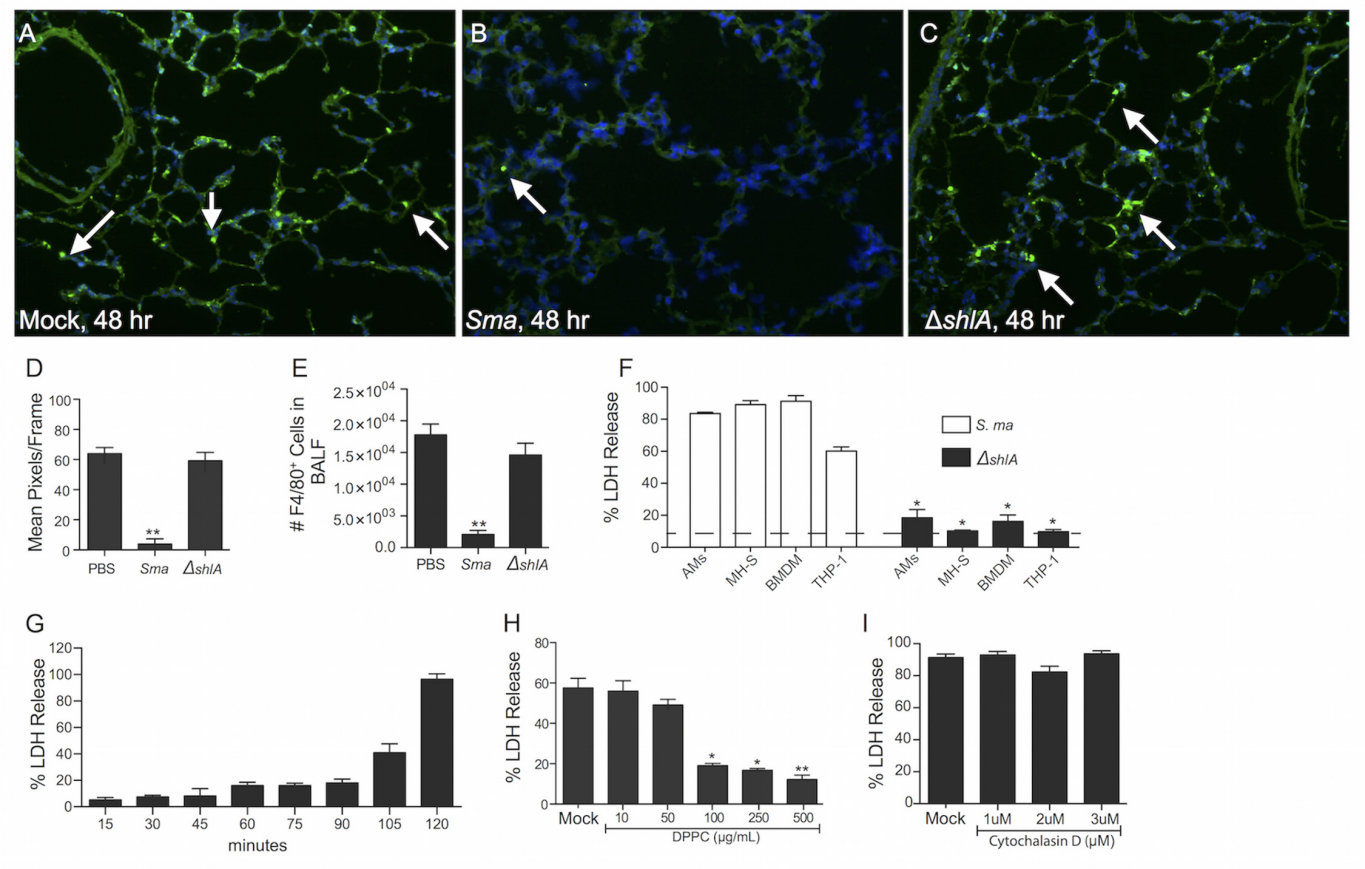
*S*. *marcescens* causes PFT-dependent cell death in macrophages. **A-C)** BALB/c mice were infected with 1.0 x 10^6^ CFU of wildtype *S*. *marcescens* (*Sma*), *S*. *marcescens* with an isogenic deletion of *shlA* (Δ*shlA*) at the same dose, or mock infected with and equal volume of PBS (Mock). Fluorescent images show macrophages (F4/80^+^, green) and all nucleated lung cells (DAPI, blue) present in lung tissue taken at 48h post-infection. (**D)** Mean pixels per frame analysis of images captured corresponding to punctate points of F4/80 (4 images per mouse, n = 4–6 mice/cohort) as determined using ImageJ64. **E)** Number of F4/80 positive cells in 50 μl BALF as determined using FACs analysis. **F)** LDH cell death assay of primary mouse AMs, mouse AM cell line MH-S, mouse bone marrow-derived macrophages (BMDM), and the human macrophage-like cell line THP-1, following infection with *Sma* or Δ*shlA*; dashed line shows baseline LDH release from uninfected cells. **G)** Kinetics of *Sma* cytotoxicity in MH-S cells as determined by LDH. **H)** LDH release assay from MH-S cells infected with *S*. *marcescens* mock or pretreated with different concentrations of dipalmitoylphosphatidylcholine (DPPC). **I)** MH-S cells pretreated with Cytochalasin D were infected with *Sma* and cytotoxicity measured using LDH release assay. For multiple group comparisons Dunn’s multiple-comparison post-test was used: *, *P* ≤ 0.05, **, *P* ≤ 0.01, ***, *P* ≤ 0.001. Data from *in vitro* experiments are representative of ≥3 separate experiments each with 8 biological replicates.

In agreement with our published report [18], we anew failed to detect F4/80^+^ cells in lung sections from mice 48h after intratracheal infection with *S*. *marcescens* (Fig 1A, 1B and 1D). In contrast, cells with strong F4/80 signal were present in lung sections from mice infected with a ShlA deficient mutant (Fig 1A, 1C and 1D). Similar results were obtained when bronchoalveolar lavage fluid (BALF) from infected mice was examined using flow cytometry (FACS) (Fig 1E). To determine the extent of macrophage susceptibility we challenged assorted macrophages *in vitro* with *S*. *marcescens*. Primary mouse AMs, the mouse AM cell line MH-S, mouse bone marrow-derived macrophages (BMDM), and the human macrophage-like cell line THP-1, all showed exquisite ShlA-dependent cytotoxicity following infection (Fig 1F). *In vitro*, macrophage death was rapid, with >80% of MH-S cells having died within 2h of challenge at an MOI of 1.0 (Fig 1G). Some PFTs can be neutralized using the lipid 2-dipalmitoylphosphatidylcholine (DPPC) [19]. Addition of DPPC protected MH-S macrophages in a dose-dependent fashion against *S*. *marcescens* (Fig 1H). Finally, inhibition of phagocytosis using cytochalasin D had no impact on MH-S killing by *S*. *marcescens* (Fig 1I). Thus, macrophages seemed to be exquisitely susceptible to some form of ShlA-induced death and the cytotoxic effects of this PFT could occur without bacterial internalization.

### Necroptosis is activated following macrophage exposure to PFT-producing pathogens

**Fig 2:**
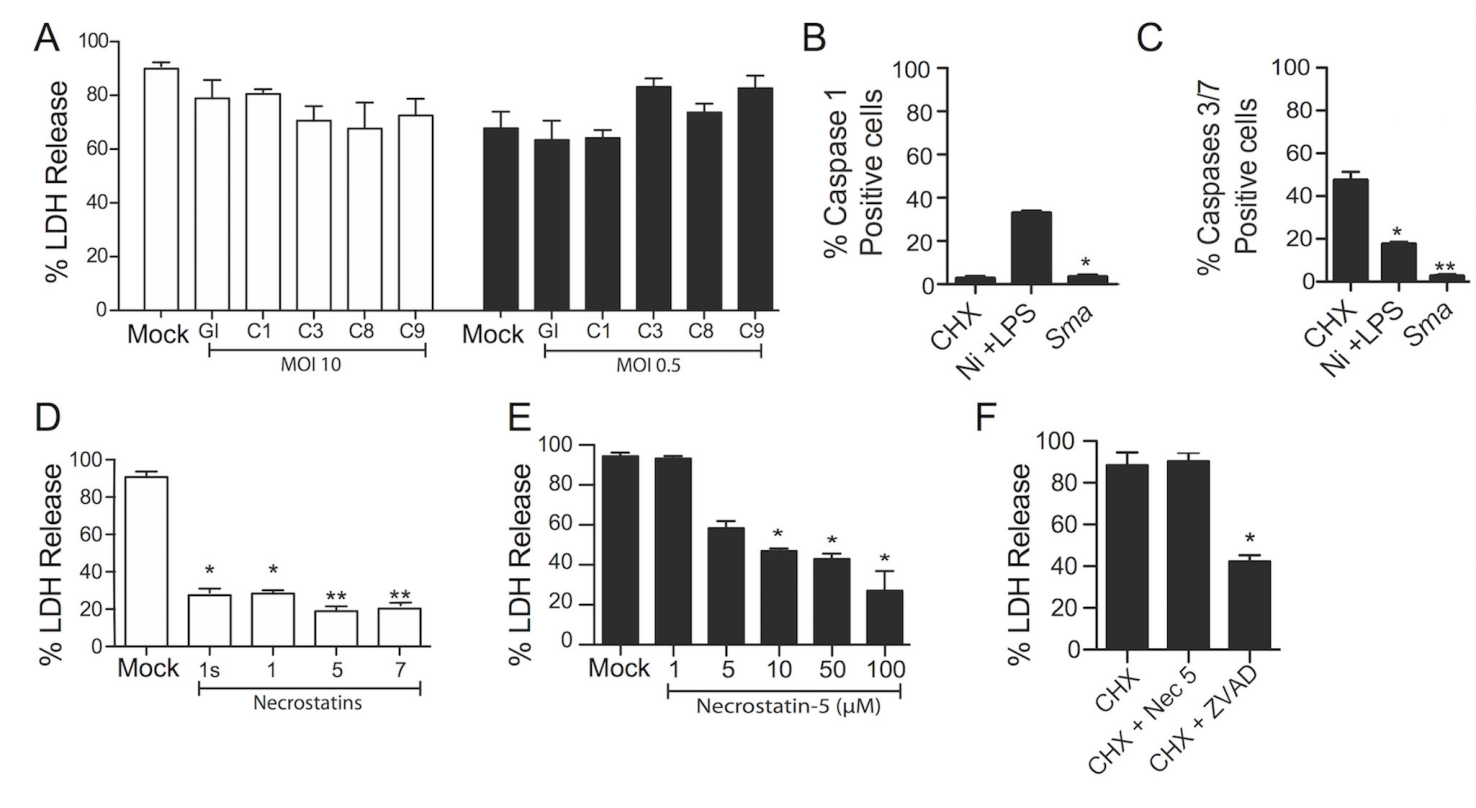
RIP1 and not caspases are required for *S*. *marcescens* induced macrophage cell death. **A)** LDH release assay of MH-S macrophages infected with a high and low MOI of *S*. *marcescens* (*Sma*) following mock or pretreatment with the general caspase inhibitor Z-VAD-FMK (GI), caspase-1 inhibitor Z-WEHD-FMK (CI) caspase-3 inhibitor Z-DEVD-FMK (C3), caspase-8 inhibitor Z-YVAD-FMK (C8), and caspase-9 inhibitor Z-LEHD-FMK (C9), all at a 10 μM concentration. Percent positive cells for **B)** caspase-1 and **C)** caspase-3/7 activity 2h after *Sma* infection following FLICA staining and measured by FACs analyses. For controls, cells with nigericin induced pyroptosis (Ni +LPS; lipopolysaccharide at 10 ng/mL for 4h then Ni at 10 μM for 6 h) and cycloheximide (CHX; 2,000 μg/ml) induced apoptosis were measured, respectively. **D)** LDH release assay of MH-S macrophages infected with *Sma* at an MOI of 1 following their mock or pretreatment with necrostatin-1s, -1, -5, or -7 at a concentration of 100 μM. **E)** LDH release assay of MH-S macrophages infected with *Sma* after pretreatment with necrostatin-5 at increasing concentrations. **F)** LDH release assay of MH-S macrophages treated with CHX after pretreatment with necrostatin-5 (Nec 5; 100 μM) or ZVAD (10 μM). For multiple group comparisons Dunn’s multiple-comparison post-test was used: *, *P* ≤ 0.05, **, *P* ≤ 0.01, ***, *P* ≤ 0.001. Data are representative of ≥3 separate experiments, each with 8 biological replicates.

We sought to determine the mechanisms that drive macrophage death following their exposure to *S*. *marcescens* [9]. We first tested a role for caspase-mediated apoptosis and pyroptosis. At both high and low MOI of *S*. *marcescens* infection pretreatment of cells with inhibitors of caspases 1, 3, 8, 9, and a general caspase inhibitor, had no effect on ShlA-induced death of MH-S cells (Fig 2A). Using FACS we determined that infection with *S*. *marcescens* did not lead to the activation of caspase-1 (Fig 2B) or caspases-3/7 (Fig 2C), further ruling out pyroptosis and apoptosis as the responsible form of cell death. Importantly, pretreatment of MH-S cells with necrostatin-1s, -1, -5, or -7, all significantly reduced cell death (Fig 2D). These treatments prevent necroptosis by their inhibition of RIP1. Protection against *S*. *marcescens* was dose-dependent and greatest when cells were pretreated with 100μM of necrostatin-5 (Fig 2E). Specificity of necrostatin-5 at 100μM was confirmed by its inability to protect against cycloheximide-induced apoptosis (Fig 2F).

**Fig 3:**
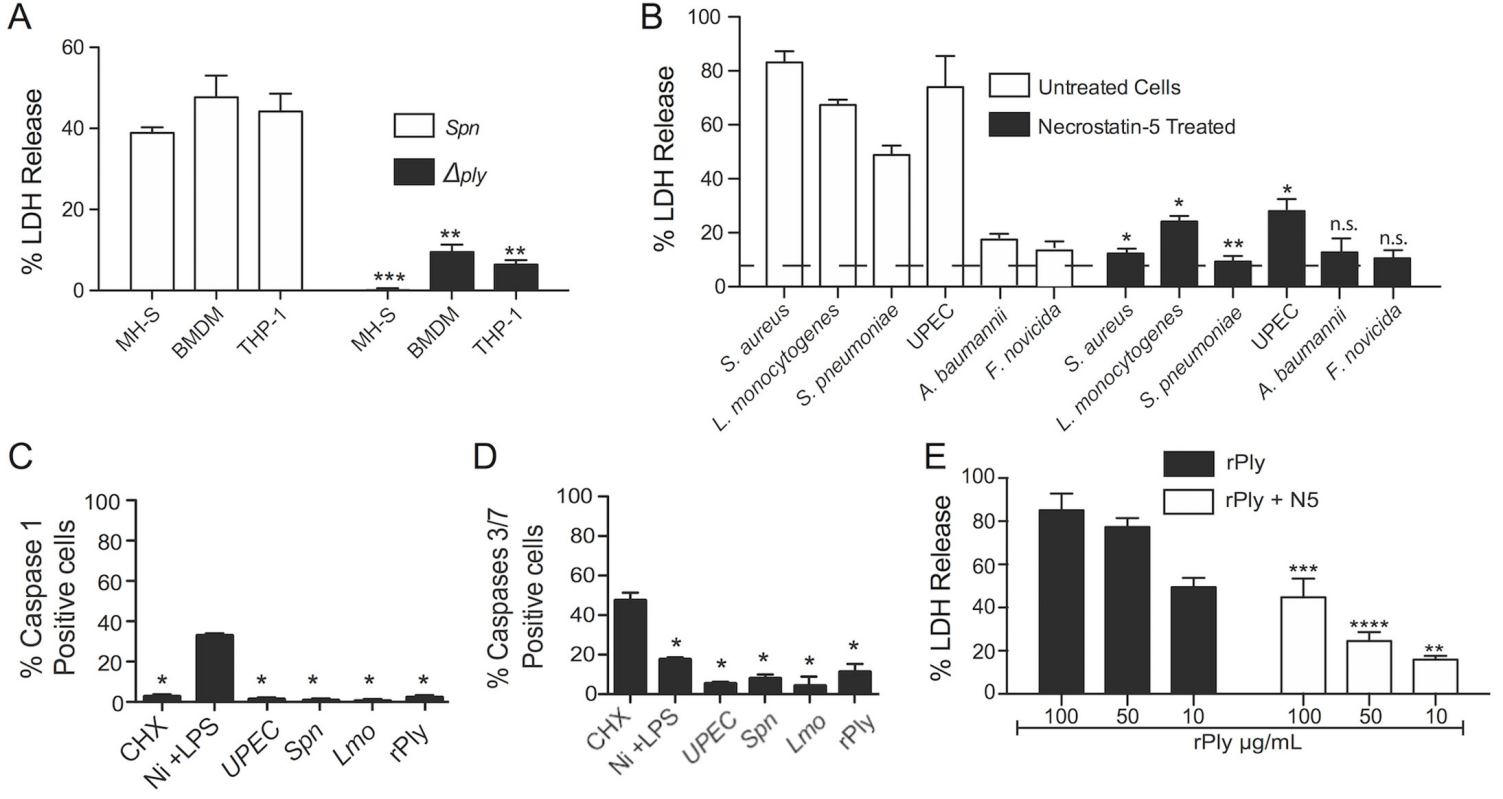
PFT-producing pathogens and pneumolysin induce RIP1 dependent macrophage cell death. **A)** LDH release assay of MH-S, BMDM, and THP-1 macrophages infected either with *S*. *pneumoniae* (*Spn*) or an isogenic mutant deficient in pneumolysin (Δ*ply*) both at MOI of 100. **B)** LDH release assay of MH-S macrophages infected with *S*. *aureus* (*Sau*) (MOI 10), *L*. *monocytogenes* (*Lmo*) (MOI 10), *S*. *pneumoniae* (MOI 100), UPEC (MOI 100), *A*. *baumannii* (MOI 100), and *F*. *tularensis novicida* (MOI 100). Black bars show LDH release when cells were pretreated with necrostatin-5 (100 μM), dashed line shows base line LDH release from uninfected cells. Percent positive cells for **C)** caspase-1 and **D)** caspase-3/7 activity 2h after UPEC, *Spn* and *Lmo* infection, or recombinant pneumolysin (rPly) challenge following FLICA staining and measurement by FACs analyses. For controls pyroptosis (Ni + LPS) and apoptosis (CHX) induced cells were included in the analyses. **E)** LDH release assay of MH-S macrophages treated with different concentrations of recombinant pneumolysin (rPly) following mock or pretreatment with necrostatin-5 at 100 μM. For multiple group comparisons Dunn’s multiple-comparison post-test was used: *, *P* ≤ 0.05, **, *P* ≤ 0.01, ***, *P* ≤ 0.001. Data are representative of ≥3 separate experiments, each with 8 biological replicates.

MH-S, BMDM, and THP-1 macrophages were also susceptible to killing by *S*. *pneumoniae*. In this instance cell death was also PFT-dependent, requiring pneumolysin, a cholesterol-dependent cytolysin (Fig 3A). As such, we tested whether diverse PFT-producing bacteria induced macrophage necroptosis. Necrostatin-5 protected MH-S cells against challenge with *S*. *aureus*, *Listeria monocytogenes*, *S*. *pneumoniae*, and uropathogenic *Escherichia coli* (UPEC) (Fig 3B). Necrostatin-5 had no effect on cells infected with the non-PFT producing bacteria *Francisella novicida* and *Acinetobacter baumannii*, albeit these induced substantially lower mortality despite a high MOI (Fig 3B). MH-S cells infected with these PFT-producing pathogens did not activate caspase-1 (Fig 3C) or caspase-3/7 (Fig 3D), again ruling out pyroptosis or apoptosis. Purified ShlA rapidly precipitates in solution and loses its cytolytic properties [20]. Instead, we tested the ability of necrostatin-5 to block cell death caused by purified recombinant pneumolysin. Pretreatment of MH-S cells with necrostatin-5 protected against pneumolysin-induced death (Fig 3E). Pneumolysin treatment did not trigger activation of caspases-1 (Fig 3C) nor -3/7 (Fig 3D), indicating that this PFT most likely killed macrophages via necroptosis.

### RIP3 and MLKL are required for PFT-induced necroptosis

**Fig 4:**
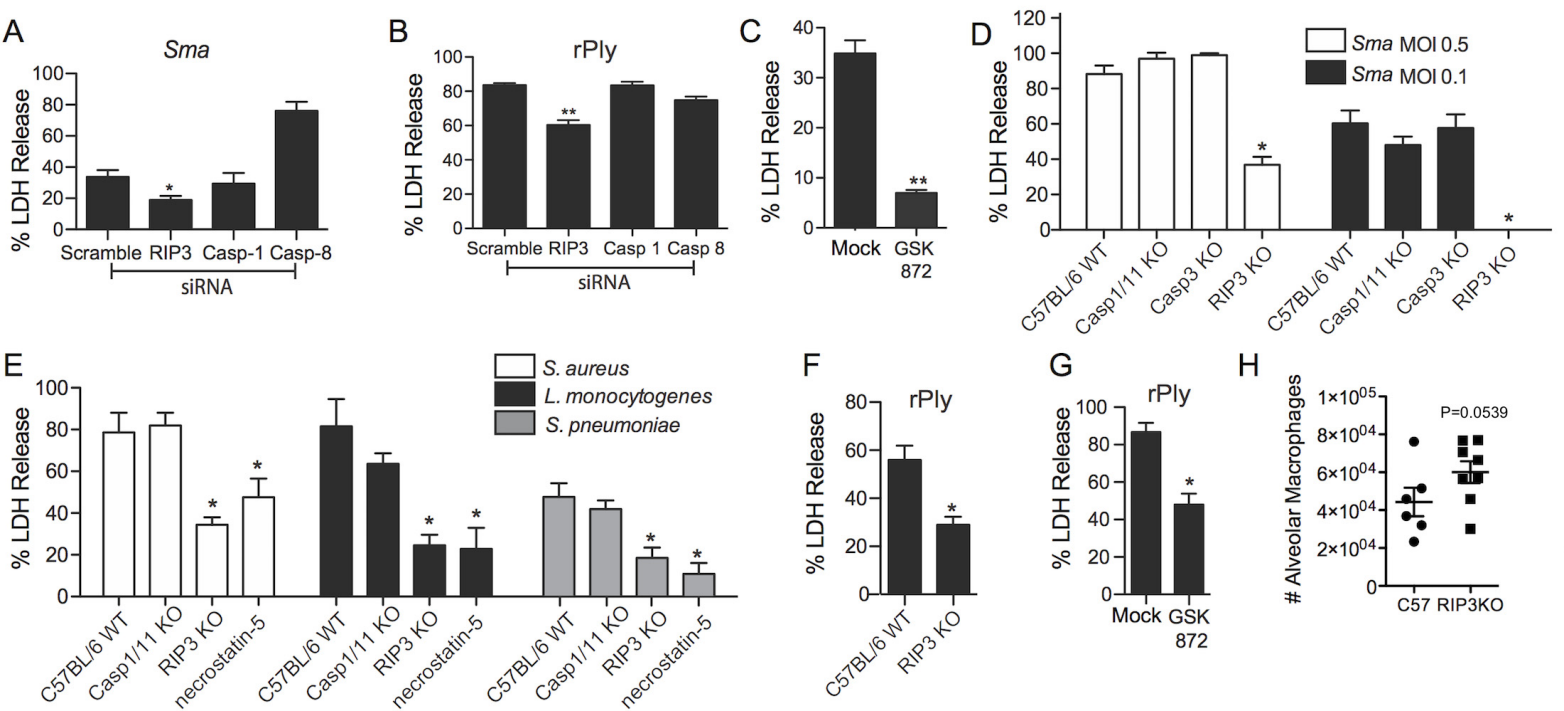
RIP3 is required for PFT induced necroptosis. **A)** LDH release assay of THP-1 cells transfected with siRNAs targeting RIP3, caspase-1 (Casp-1), caspase-8 (Casp-8) and a scramble control, infected with (A) *Sma* or challenged with **B)** recombinant pneumolysin (rPly). **C**) LDH release assay of MH-S macrophages infected with *Sma* at an MOI of 0.1 mock or following pretreatment with RIP3 inhibitor GSK’872. **D)** LDH release assay of BMDM from WT-C57BL/6, Caspase 1/11 KO, Caspase 3 KO and RIP3 KO mice, infected with *Sma* at an MOI of 0.5 or 0.1. **E)** LDH release of BMDM from wildtype mice, Caspase 1/11 (Casp1/11) KO, RIP3 KO, and BMDM from wildtype mice pretreated with necrostatin-5 (100μM) following their infection with *S*. *aureus* (MOI 10), *L*. *monocytogenes* (MOI 10) and *S*. *pneumoniae* (MOI 100). **F)** LDH release assay of BMDM from wild type C57BL/6 or RIP3 KO mice following their challenge with recombinant pneumolysin (rPly). **G)** LDH release assay of MH-S macrophages challenged with rPly with and without pretreatment with GSK’872 (10 μM). **H)** Alveolar macrophage numbers in BALF of RIP3 KO mice 18h after intratracheal infection with *S*. *marcescens*. Mann-Whitney tests were applied for two-group comparisons, for multiple group comparisons Dunn’s multiple-comparison post-test was used: *, *P* ≤ 0.05. Data are representative of ≥3 separate experiments, each with 8 biological replicates.

To further implicate necroptosis and rule out other forms of cell death, THP-1 cells were transfected with siRNA against RIP3, caspase-1, caspase-8, or with control siRNA, and infected with *S*. *marcescens* or challenged with pneumolysin. Only THP-1 cells that received siRNA against RIP3 showed partial protection against cell death (Figs 4A, 4B and S1A). Pre-treatment with GSK’872, a selective mouse RIP3 inhibitor [3], almost completely blocked MH-S death following *S*. *marcescens* infection (Fig 4C). BMDM from RIP3 KO mice were also resistant to death when compared to BMDM from wildtype mice (Fig 4D). In contrast, no protection was observed for BMDM from mice deficient in caspases-1/11 or caspase-3 (Fig 4D). Protection against death was also observed when BMDM from RIP3 KO mice were challenged with *S*. *aureus*, *L*. *monocytogenes*, and *S*. *pneumoniae*. The level of protection was equivalent to that observed when wildtype BMDM were pretreated with necrostatin-5 (Fig 4E). Protection against purified pneumolysin was also observed in BMDM from RIP3 KO mice (Fig 4F) and in BMDM from wildtype mice treated with GSK’872 (Fig 4G). Finally, RIP3 KO mice infected with *S*. *marcescens* had a strong trend (*P* = 0.0539) towards a higher mean number of F480^hi^, CD11c^+^ cells (i.e. alveolar macrophages) in the BALF 18h post-infection (Fig 4H).

**Fig 5:**
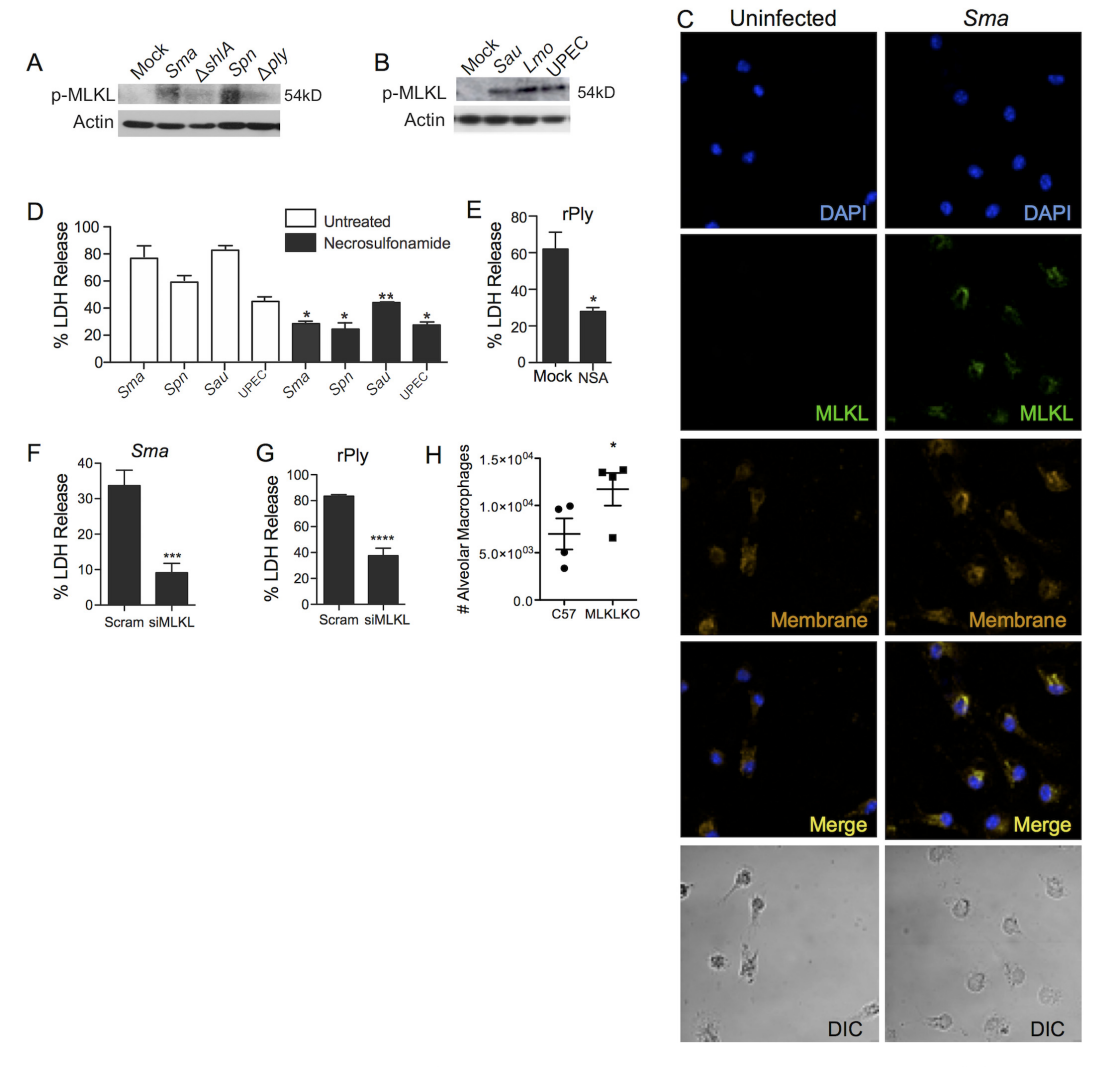
MLKL is required for PFT induced necroptosis. Western blot for pMLKL in MH-S whole cell lysates following their challenge with **A)** *S*. *marcescens* (*Sma*), Δ*shlA*, *S*. *pneumoniae* (*Spn*) and Δ*ply* and **B)** *S*. *aureus* (*Sau*), *L*. *monocytogenes* (*Lmo*) and UPEC. **C)** Immunofluorescence for MLKL (Green) and lipid membrane (Red) in uninfected and *Sma* infected MH-S cells. Nuclei were stained with DAPI (blue). **D)** LDH release assay of infected THP-1 macrophages following mock or pretreatment with the MLKL inhibitor necrosulfonamide (100μM). MOIs were: *Sma =* 1,*Spn* = 100, Sau = 10, UPEC = 100. **E)** LDH release assay of THP-1 macrophages following challenge with recombinant pneumolysin (rPly), with and without pretreatment with necrosulfonamide (NSA, 100μM, black bars). LDH release assay of **F)** *Sma* infected or **G)** rPly challenged THP-1 macrophages transfected with siRNA targeting MLKL. As control scrambled (Scram) siRNA was used. **H)** Alveolar macrophage numbers in BALF of MLKL KO mice 18h after intratracheal infection with *S*. *marcescens*. Student *t-*tests were applied for two-group comparisons, for multiple group comparisons Dunn’s multiple-comparison post-test was used: *, *P* ≤ 0.05. Data are representative of ≥3 separate experiments, during LDH assays each with 8 biological replicates.

MLKL is the effector of necroptosis and is activated by the necroptosome; with MLKL being phosphorylated (pMLKL) and then its targeting of cell membranes [2]. Cell lysates from MH-S cells infected with *S*. *marcescens* and *S*. *pneumoniae* showed increased levels of pMLKL in a PFT-dependent manner when examined by Western blot (Fig 5A). pMLKL was also present in MH-S cells infected with wildtype *S*. *aureus*, *L*. *monocytogenes*, and UPEC (Fig 5B). Challenge of MH-S cells with *S*. *marcescens* resulted in the co-localization of MLKL at the plasma membrane (Fig 5C). *S*. *pneumoniae* and recombinant pneumolysin also caused what appeared to be membrane localization of MLKL (S2A Fig). The distribution of MLKL in challenged cells was consistent with that observed when cells underwent TNFα/ZVAD induced necroptosis (S2B Fig). Inhibition of MLKL in human macrophages with necrosulfonamide protected against death caused by *S*. *marcescens*, *S*. *pneumoniae*, *S*. *aureus*, and UPEC (Fig 5D). Necrosulfonamide also protected against pneumolysin-mediated death (Fig 5E). Accordingly, immunofluorescent studies showed that necrosulfonamide prevented MLKL aggregation at the plasma membrane of challenged THP-1 cells (S2C Fig). In this instance MLKL seemed to be increased over the control and diffuse throughout the cytoplasm. THP-1 cells transfected with siRNA targeting MLKL were protected against both *S*. *marcescens* (Figs 5F and S1B) and recombinant pneumolysin (Fig 5G). Finally, the number of alveolar macrophages detected in the BALF of infected MLKL KO mice 18h post-infection was significantly higher than wild type controls (Fig 5H). Thus, we conclude that PFTs produced by diverse Gram-negative and Gram-positive pathogens rapidly induce macrophage necroptosis and this can be blocked with inhibitors of RIP1, RIP3, and MLKL.

### NLRP3 and ASC are linked to PFT-induced necroptosis in macrophages

**Fig 6:**
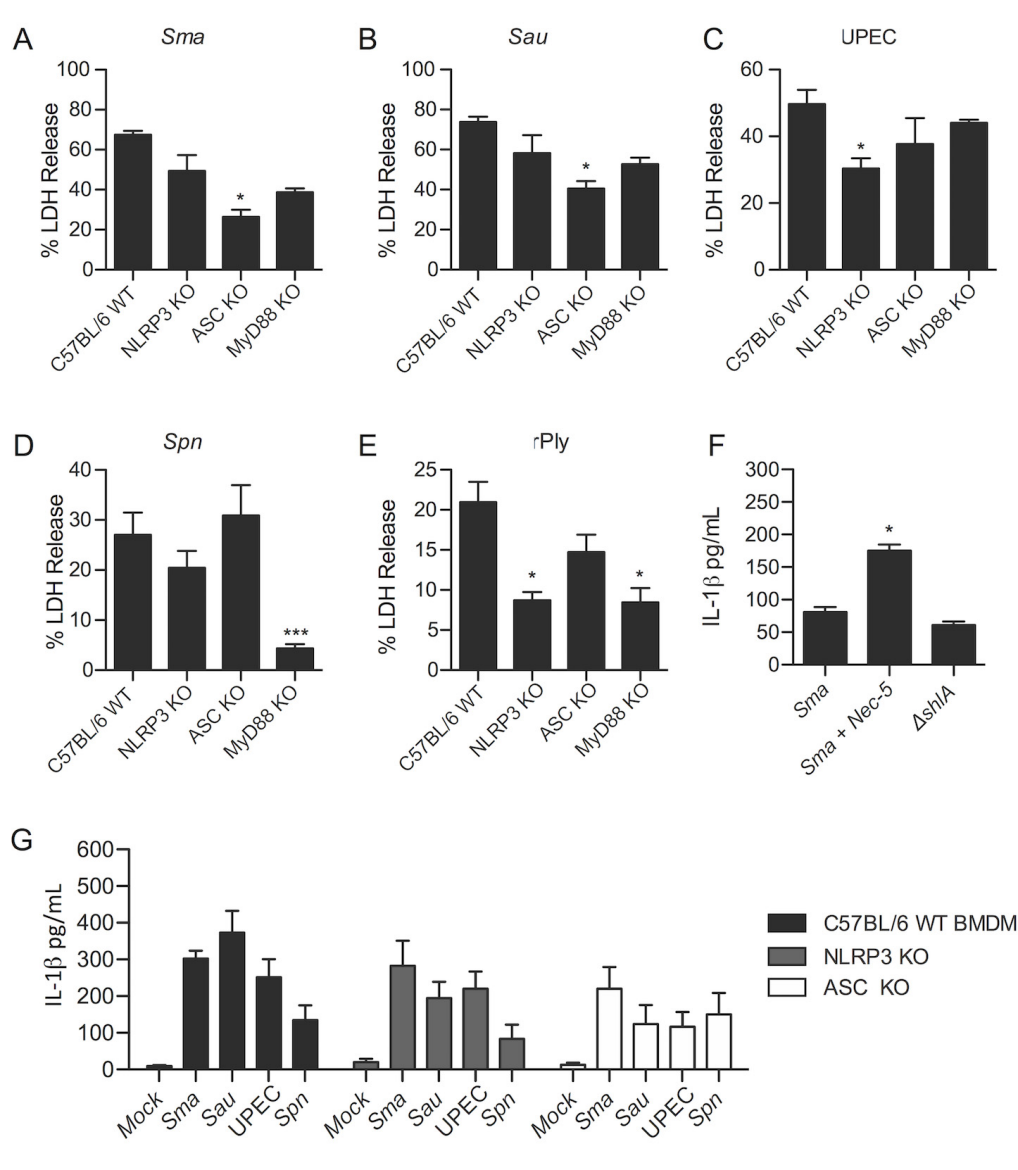
Inflammasome components can be linked to PFT-induced necroptosis in macrophages. LDH release assay of BMDM from C57BL/6 wildtype (WT), NLRP3 KO, ASC KO and MyD88 KO mice, infected with **A)** *S*. *marcescens* (*Sma*; MOI = 0.5), **B)** *S*. *aureus* (*Sau*; MOI = 10), **C)** UPEC (MOI = 100), **D)** *S*. *pneumoniae* (Spn; MOI = 100) and challenged with **E)** recombinant pneumolysin (rPly; 100 μg/ml). **F)** Levels of IL-1β detected by ELISA in supernatants from MH-S macrophages infected with *Sma* or Δ*shlA*. Necrostatin-5 (Nec-5) was added at 100μM. **G)** Levels of IL-1β detected in supernatants from WT, NLRP3 KO, ASC KO BMDMs infected with *Sma*, *Sau*, UPEC and *Spn* and uninfected controls. For multiple group comparisons Dunn’s multiple-comparison post-test was used: *, *P* ≤ 0.05. Data are representative of ≥3 separate experiments, each with 8 biological replicates.

We infected BMDM from C57BL/6 wildtype mice, NLRP3 KO, MyD88 KO, and ASC KO mice with our panel of bacteria and then measured cytotoxicity. For *S*. *marcescens* and *S*. *aureus*, BMDM lacking ASC showed partial protection (Fig 6A and 6B). For UPEC and pneumolysin challenged BMDM, those lacking NLRP3 were protected (Fig 6C and 6E). Finally, the absence of MyD88 protected cells against both live *S*. *pneumoniae* and recombinant pneumolysin (Fig 6D and 6E). We conclude these upstream inflammasome and TLR signaling components participate in PFT-activated cell death, albeit not through the activation of caspase-1 (Figs 3C, 4C and 4E). Infection of MH-S cells with *S*. *marcescens* resulted in the detection of IL-1β in culture supernatants. This was not ShlA dependent. In fact, pretreatment of BMDM with necrostatin-5 enhanced levels of detectable IL-1β (Fig 6F). Infection of wildtype BMDM with other pathogens also resulted in detectable IL-1β in culture supernatants. This also occurred in BMDM from NLRP3 and ASC KO mice (Fig 6G). Thus, caspase-1 independent release of IL-1β occurred during bacterial infection of macrophages and RIP1 activation was at least in one instance suppressive of this.

### PFT-induced necroptosis is triggered by ion loss and mitochondrial damage

**Fig 7:**
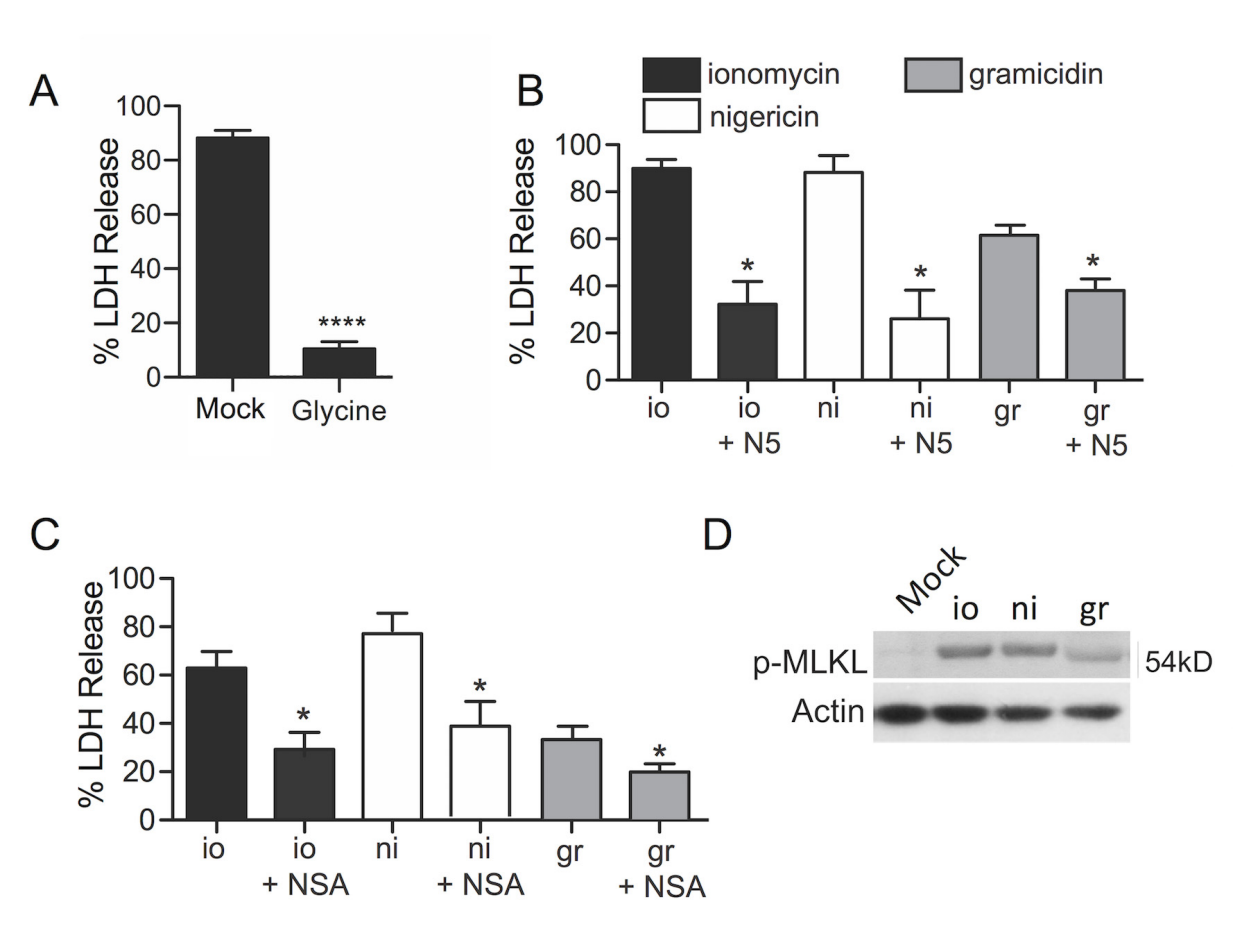
Ion loss is sufficient to induce macrophage necroptosis. **A)** LDH release cytotoxicity assay of MH-S macrophages mock or pretreated with glycine (2 mM) then infected with *S*. *marcescens*. **B)** LDH release cytotoxicity assay of MH-S macrophages following their treatment with ionomycin (io; 20μM), nigericin (ni; 20μM) and gramicidin (gr; 20μM) for 6 hours, with and without pretreatment with necrostatin-5 (N5; 100μM). **C)** LDH release cytotoxicity assay of THP-1 macrophages treated with io, ni, and gr, with and without pretreatment with necrosulfonamide (NSA; 100μM). **D)** Western blot for pMLKL in whole cell lysates from MH-S macrophages following their treatment with io, ni, gr. Mann-Whitney U tests were applied for two-group comparisons, for multiple group comparisons Dunn’s multiple-comparison post-test was used: *, *P* ≤ 0.05, **, *P* ≤ 0.01, ***, *P* ≤ 0.001. Data are representative of ≥3 separate experiments, each with 8 biological replicates (during LDH assays).

Given that membrane disruption by PFTs results in dysregulated ion flux, we sought to determine if loss of membrane potential was sufficient to induce necroptosis. Cells treated with glycine, which blocks membrane permeabilization [21], were protected against *S*. *marcescens* (Fig 7A). A role for ion dysregulation independent of membrane damage was supported by our finding that macrophages treated with the ionophores ionomycin, which is selective for Ca^++^ intake [22]; nigericin, which is selective for K^+^ loss [23]; and gramicidin, a pore-forming ionophore permissive for inorganic monovalent cations (e.g. Na^+^); all showed a form of cell death that could be blocked by inhibition of RIP1 (Fig 7B) or MLKL (Fig 7C). Immunoblots of cell lysates showed the *de novo* presence of pMLKL in lysates from ionophore treated cells (Fig 7D). Membrane association of MLKL was detected in ionophore-treated cells by immunofluorescence and could be blocked with necrostatin-5 (S3 Fig).

**Fig 8:**
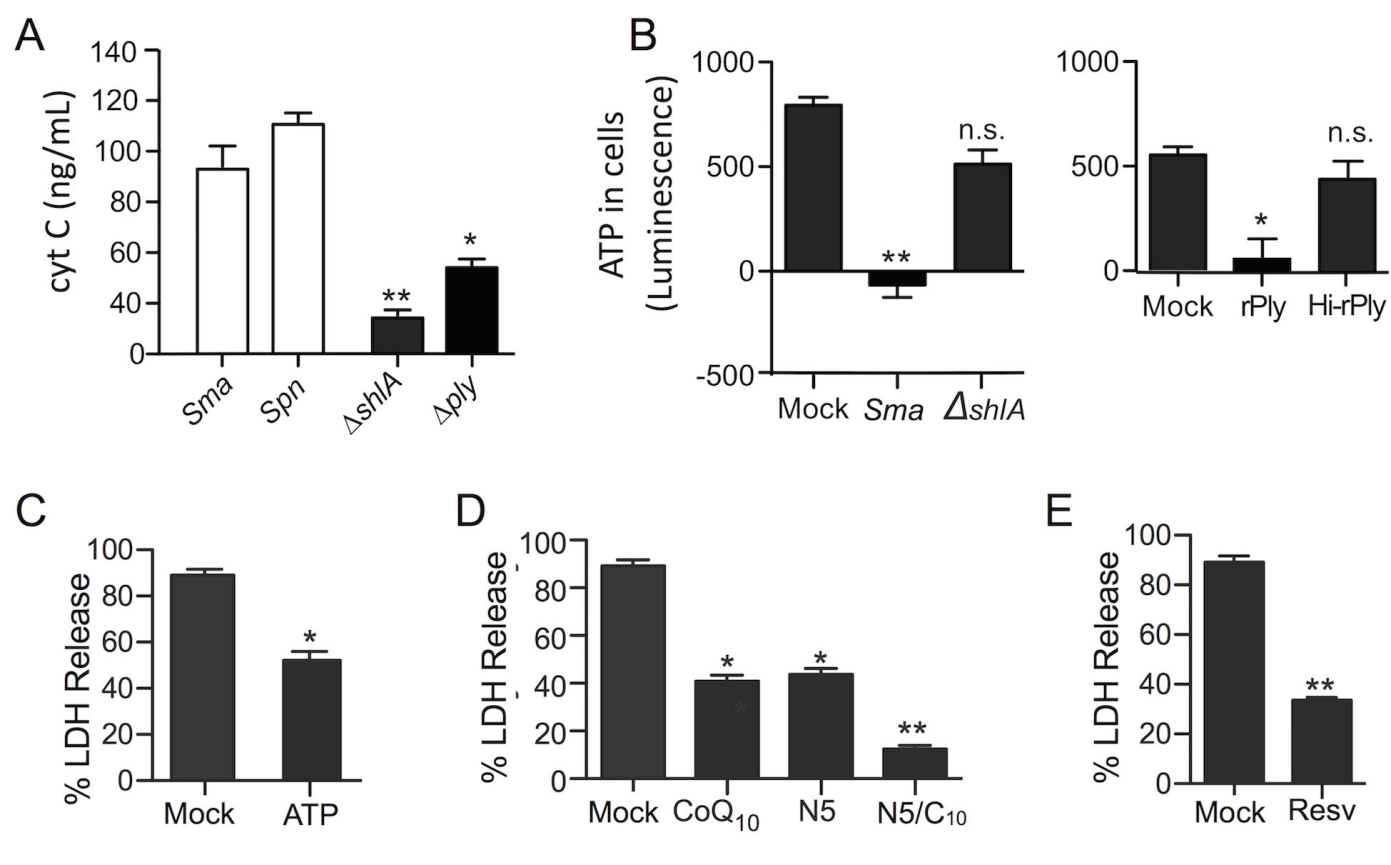
Mitochondrial damage and ATP depletion are required during PFT-induced necroptosis. **A)** Cytochrome C (cyt C) levels in culture supernatants of MH-S cells infected with *S*. *marcescens* (*Sma*), *S*. *pneumoniae* (*Spn*), Δ*shlA*, and Δ*ply*. **B)** ATP levels in MH-S cell lysates following their infection with *Sma*, Δ*shlA*, and recombinant pneumolysin (rPly) or heat-inactivated rPly (HI-rPly) as measured using a luminescent cell viability assay. LDH release assay of MH-S cells infected with *Sma* following mock or pretreatment with **C)** soluble ATP (10 μg/mL), **D)** CoQ_10_ (100μM), necrostatin-5 (N5; 100μM), N5/C_10_ (100μM each), or **E)** resveratrol (Resv; 100μM). In vitro experiments were done at an MOI of 1 and 100 for *Sma* and *Spn*, respectively. Mann-Whitney U tests were applied for two-group comparisons, for multiple group comparisons Dunn’s multiple-comparison post-test was used: *, *P* ≤ 0.05, **, *P* ≤ 0.01, ***, *P* ≤ 0.001. Data are representative of ≥3 separate experiments; during LDH assay each with 8 biological replicates.

Since PFTs can damage the mitochondrial membrane both directly and indirectly [24], we explored whether mitochondrial damage and dysfunction during PFT challenge lead to macrophage necroptosis. MH-S cells infected with wildtype but not PFT-deficient *S*. *marcescens* or *S*. *pneumoniae* had increased levels of cytochrome C in their supernatants, an indicator of mitochondrial damage (Fig 8A). Significantly lower total levels of ATP were also observed in *S*. *marcescens* infected and pneumolysin exposed MH-S cells (Fig 8B). MH-S death could be blocked by the addition of soluble ATP (Fig 8C) and by pretreatment of macrophages with co-enzyme Q_10_ (CoQ_10_) (Fig 8D)_,_ which enhances ATP production [25]. Resveratrol, which increases mitochondria numbers [26], was also protective against challenge (Fig 8E). Importantly, necrostatin-5 together with CoQ_10_ (N5/C_10_) had an additive and almost complete protective effect against cell death (Fig 8D). It is worth noting that addition of ATP alone did not prevent MLKL localization to the membrane, however pretreatment with CoQ_10_ did (S4 Fig). These results implicate low levels of ATP as a result of mitochondrial damage as a signal for PFT-induced necroptosis and suggest that CoQ_10_ has beneficial effects that are in addition to enhanced ATP production.

**Fig 9:**
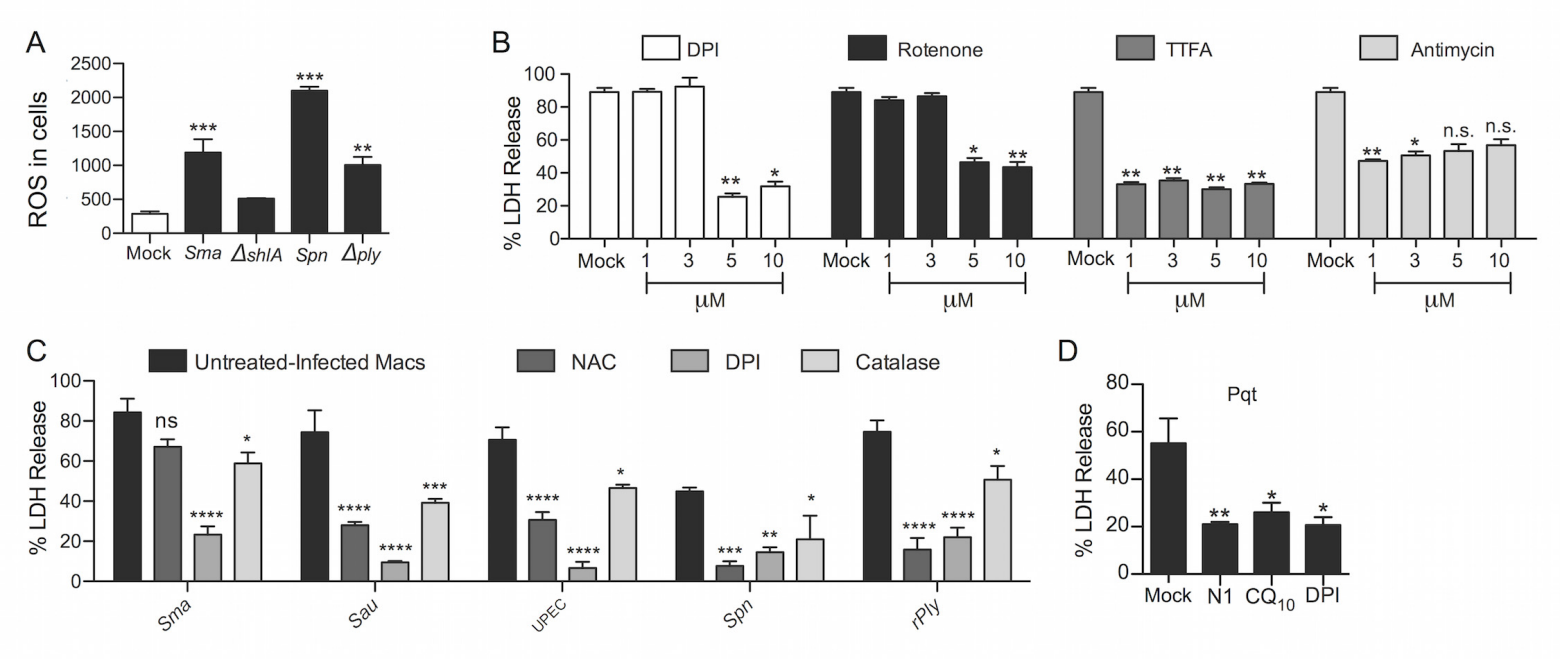
Reactive oxygen species mediate PFT induced necroptosis in macrophages. **A)** Levels of ROS in MH-S cells infected with *S*. *marcescens* (*Sma*), Δ*shlA*, *S*. *pneumoniae* (*Spn*), or Δ*ply* as measured using the indicator H2-DCF. **B)** LDH release cytotoxicity assay of MH-S macrophages infected with *Sma* following pretreatment with the general ROS inhibitor DPI, complex I ROS inhibitor rotenone, complex II ROS inhibitor TTFA, and complex III ROS inhibitor antimycin at the designated concentrations. **C)** LDH release cytotoxicity assay of MH-S macrophages infected with *Sma*, *S*. *aureus* (*Sau*), UPEC, *Spn*, or challenged with recombinant pneumolysin (rPly) following mock or pretreatment with n-acetyl cysteine (NAC; 100 μM), DPI (10 μM), and with catalase (10μM), respectively. (D) LDH release cytotoxicity assay of MH-S macrophages treated with ROS inducer paraquat (pqt) at a concentration of 500uM for 12h following mock or pretreatment with necrostatin-1 (N1, 100μM), CoQ_10_ (100μM) or DPI (10 μM). Mann-Whitney U tests were applied for two-group comparisons, for multiple group comparisons Dunn’s multiple-comparison post-test was used: *, *P* ≤ 0.05, **, *P* ≤ 0.01, ***, *P* ≤ 0.001. Data are representative of ≥3 separate experiments, each with 8 biological replicates.

During TNFα/ZVAD induced necroptosis, ROS from mitochondrial complexes 1 and 2 has been reported to serve as an initiating signal for cell death execution [27]. Yet other investigators have reported that antioxidant treatment does not suppress necroptosis [28, 29]. To explore the role of ROS in PFT-induced macrophage necroptosis, we first confirmed that *S*. *marcescens* and *S*. *pneumoniae* induce ROS in a PFT-dependent manner in MH-S cells (Fig 9A). Subsequently, we observed that the general ROS inhibitor diphenyleneiodonium (DPI) diminished *S*. *marcescens* triggered cell death (Fig 9B). The use of ROS inhibitors specific to mitochondrial complexes 1, 2 and 3, also protected against death caused by *S*. *marcescens* (Fig 9B). The general ROS inhibitor DPI, catalase, and N-acetyl cysteine (NAC) all decreased cell death induced by *S*. *marcescens*, *S*. *aureus*, UPEC, *S*. *pneumoniae* and recombinant pneumolysin (Fig 9C). Finally, generation of ROS using paraquat triggered MH-S cell death that could be blocked by inhibition of RIP1 and pretreatment with CoQ_10_ or DPI (Fig 9D). Thus, our collective results indicate that macrophages undergo PFT-mediated necroptosis due to changes in cellular ion levels and direct or indirect mitochondrial damage that leads to ATP depletion and the generation of ROS.

### Inhibition of necroptosis improves *S*. *marcescens* pneumonia outcomes

**Fig 10:**
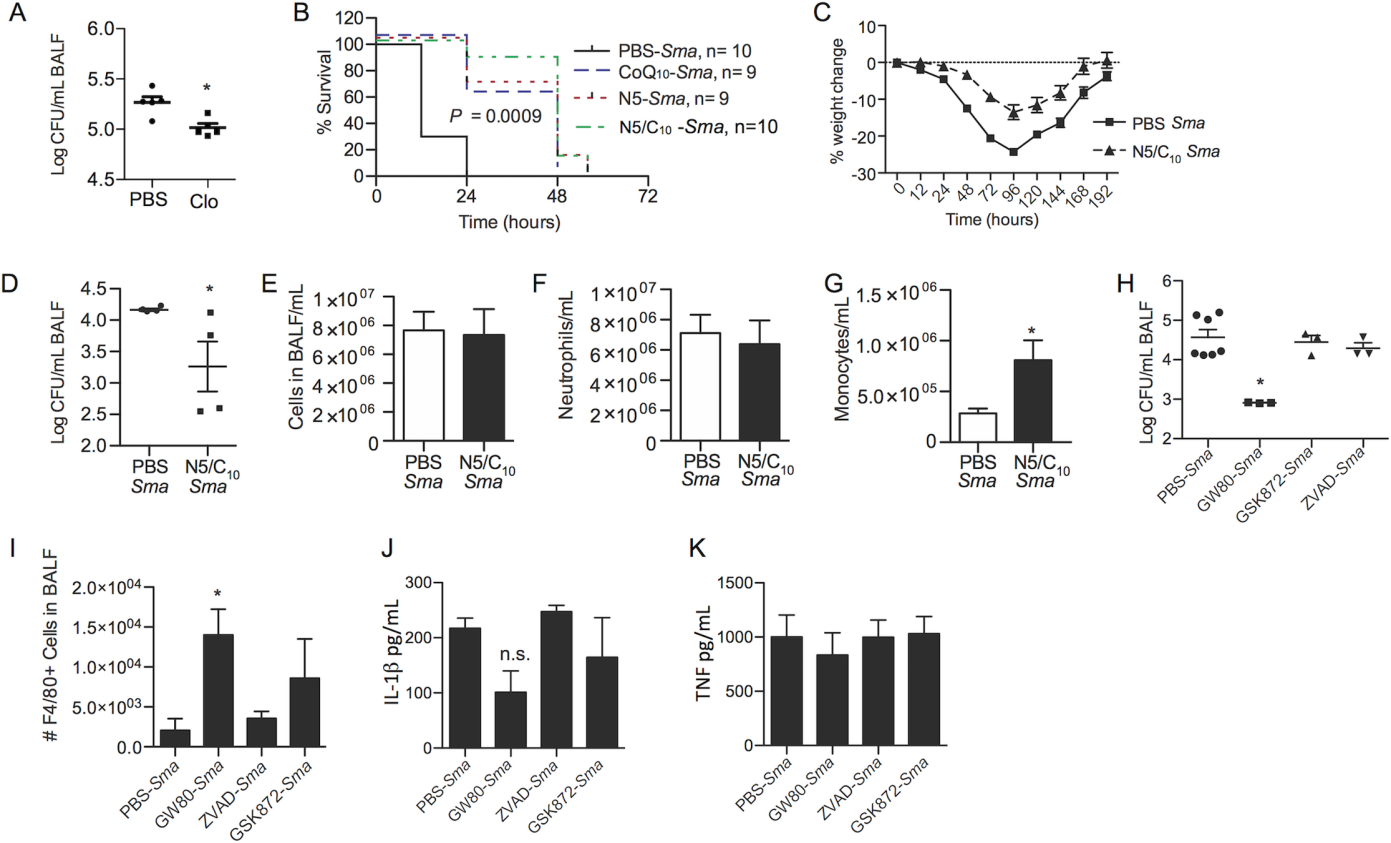
Inhibition of RIP1 or MLKL decreases morbidity and mortality during *S*. *marcescens* hemorrhagic pneumonia. BALB/c mice were infected intratracheally with *Sma* at high dose (5.0 x 10^6^ CFU) or low dose (1.0 x 10^6^ CFU). **A)** CFU recovered from the BALF of BALB/c mice pretreated with PBS or clodronate liposomes 24h after infection with low dose *Sma*. Each symbol represents an individual mouse. **B)** Survival of mice infected with the high dose of *Sma* that received intraperitoneal pre-treatment with CoQ_10,_ necrostatin-5 (N5), or N5 along with CoQ_10_ (N5/C_10_). Mice received 100 μl of a 100 μM solution of each drug intraperitoneally from time of challenge every 4h for the first 12h post-infection. **C)** Percent weight change and **D)** airway bacterial burden of *Sma* infected mice (n = 4–5/cohort) (low dose) when treated with PBS or the described N5/C_10_ therapy. The concentration of **E)** total leukocytes, **F)** neutrophils, and **G)** monocytes (*y-*axis is log scale) in Hema-3-stained cytospins of BALF from *Sma* infected mice (low dose) that were received mock or N5/C_10_ therapy. **H)** Airway bacterial burden, **I)** number of F4/80 positive cells in 50 μl, **J)** IL-1β levels, and **K)** TNFα levels in BALF from *Sma* infected mice (low dose) that received treatment with PBS, GW806742X (GW80; 100 μl of 100 μM), GSK’872 (100 μl of 10 μM) or ZVAD (100 μl of 10 μM). Mann-Whitney U tests were applied for two-group comparisons, for multiple group comparisons Dunn’s multiple-comparison post-test was used: *, *P* ≤ 0.05, **, *P* ≤ 0.01, ***, *P* ≤ 0.001. Data are representative of ≥3 separate experiments, each with 8 biological replicates.

To determine the importance of AMs on the outcome of *S*. *marcescens* pneumonia we depleted these cells in mice prior to infection using clodronate-liposomes [7]. Mice lacking AMs had decreased numbers of recoverable *S*. *marcescens* in BALF following challenge (Fig 10A). This suggests AM necroptosis was detrimental to the host. Along such lines, mice treated with necrostatin-5, CoQ_10_, or both (N5/C_10_), 2h before challenge with a lethal dose of *S*. *marcescens* and every 4h thereafter until 12h, had significantly prolonged survival (Fig 10B). In mice infected with a sublethal dose of *S*. *marcescens*, those that received a similar regimen of N5/C_10_ lost less weight than mock treated animals, indicating disease severity was attenuated (Fig 10C). At 48h post infection, bacterial titers were significantly lower in N5/C_10_ treated mice compared to controls (Fig 10D). Histological analysis showed less hemorrhage, edema, peribronchial and perivascular inflammation, and alveolar thickening in lungs of N5/C_10_ treated mice (S5A Fig). This was consistent with the reduced lung damage observed in infected MLKL KO mice (S5B Fig). Enumeration of nucleated cells in BALF showed no differences in total leukocyte numbers and neutrophils in the lungs of control versus N5/C_10_ treated infected mice. Yet, the number of monocytes was significantly greater in the BALF of mice that received N5/C_10_ (Fig 10E, 10F and 10G), indicating their protection by this treatment and consistent with prior results using RIP3 KO (Fig 4H) and MLKL KO (Fig 5H) mice. Mice that received N5/C_10_ also had a stark and visible to the eye reduction in the number of red blood cells present in collected BALF (S5C Fig), a direct indication that the severity of hemorrhagic pneumonia had been reduced.

To further investigate necroptosis inhibitors as therapeutics for *S*. *marcescens* pneumonia we also treated mice with the RIP3 inhibitor GSK’872, an MLKL inhibitor GW806742X, and the general caspase inhibitor Z-VAD. Our results showed a reduction in bacterial titers in mice that received GW806742X but not GSK’872 or Z-VAD (Fig 10H). FACS analysis of BALF showed significant differences in the number of F4/80^+^ cells detected between the GW806742X treated mice and the untreated control but not for GSK’872 or Z-VAD treated mice versus controls (Fig 10I). Of note, IL-1β and TNFα levels were unaffected by these treatments (Fig 8J and 8K). Thus, chemical inhibition of necroptosis, in particular via blockage of RIP1 and MLKL along with the support of mitochondrial function, enhanced survival of macrophages *in vivo*, diminished bacterial burden in the lungs, and lessened the pathological effects related to *S*. *marcescens* hemorrhagic pneumonia.

## Discussion

PFTs are one of the most ubiquitous types of virulence factors found in bacteria [9]. However, much remains to be learned about their mechanisms of action. Until just recently very limited evidence existed that PFTs induced necroptosis [11, 30]. The first strong indication being a recent study by Kitur and colleagues who demonstrated that during *S*. *aureus* pneumonia, several of its toxins triggered necroptosis and this meaningfully contributed to lung damage [7]. Herein, we provide independent confirmation that necroptosis is a key cell death pathway evoked during bacterial infection. What is more, we show that diverse PFT-producing pathogens including Gram-negative or -positive, extracellular or intracellular, share the ability to induce necroptosis of macrophages. We conclude that necroptosis is the principle cell death pathway triggered in macrophages by sub-lytic but still lethal levels of a PFT. This finding has broad implications and suggests that the necroptosis pathway might be targeted for clinical intervention during bacterial infections. We also report that lung damage during *S*. *marcescens* pneumonia is in part the result of rapid depletion of alveolar macrophages by necroptosis. This is a new facet in our understanding of *S*. *marcescens* pathogenesis. The fact that pneumolysin also rapidly kills alveolar macrophages suggests other PFT-producing pathogens likely do the same and this is a key first step for pathogenesis. Importantly, the ability to induce necroptosis is not the only mechanism of action for PFTs. Other described activities for these toxins presumably continue in parallel and impact pathogenesis in a cell type and anatomical specific manner [9].

Our results also provide insight as to how necroptosis might be initiated under pathogen free conditions, an area of understanding that remains unclear [5, 27]. ShlA-mediated cytotoxicity could be blocked with DPPC, the major lipid constituent of pulmonary surfactant, indicating that a PFTs interaction with the cell membrane was critical and surfactant is a key molecule to counter PFT action in the airway. Yet our results with ionophores showed that macrophage necroptosis could be induced without membrane damage [22]. Thus, membrane perturbation *per se* was not a requirement for PFT-mediated activation of necroptosis and instead some manner of ionic homeostatic disruption serves as the primary signal.

Disruption of ion homeostasis is known to cause the release of cell death signals from mitochondria and depolarization that impairs their ability to produce ATP [31, 32]. Pore-formation at the cell membrane, and subsequent ion dysregulation, may therefore indirectly damage mitochondria. PFTs have also been co-localized with mitochondrial membranes, suggesting that direct damage is also possible during infection [24]. Our observation of cytochrome C in culture supernatants as well as decreased cellular ATP levels suggests that at least one of these mitochondrial-damaging events occurs following PFT-exposure. It is important to note that we were able to protect macrophages from PFT-induced death by support of mitochondrial function with exogenous ATP, CoQ_10_, and resveratrol treatment. We also observed that PFTs triggered the generation of ROS, and that ROS caused necroptotic death of macrophages. Thus, ATP depletion and ROS are necroptosis-initiating signals induced by the mitochondrial damage caused by PFTs. Importantly, how the necroptosome senses low levels of ATP or ROS remains unknown. Of note, the ROS observed after PFT exposure was likely from damaged mitochondria, yet we cannot rule out a contribution from cellular NOXes [33].

Recently, Tait et al. showed that TNFα-induced necroptosis was not affected by mitochondrial depletion and that mitochondrial ROS production occurred in parallel but did not cause necroptosis. More specifically, and using endothelial and fibroblast cell lines, Tait et al. showed that NAC did not influence cell death caused by TNFα and the caspase inhibitor ZVAD [29]. In contrast, we show that ROS neutralized by NAC, catalase, and DPI, all decreased PFT-induced death in macrophages. This discrepancy is most likely due to the different cell types tested and type of insult, where we relied on a single agent that directly damaged the plasma membrane and mitochondria of macrophages, and Tait et al. relied on the activation of a death receptor and the suppression of normal caspase signaling. Thus, the upstream signaling cascades that lead to necroptosis are likely different under these two conditions. It is worth noting, that Koo et al., report that inhibition of oxidative phosphorylation within mitochondria leads to activation of the necroptosome within lung epithelial cells [34]. This report further supports the notion that necroptosis signaling may vary depending on cell type and the form of cell insult. Along such lines, Wang et al. found that RNA viruses trigger NLRP3 inflammasome through a RIP1-RIP3-DRP1 signaling pathway [35].

PFTs have been shown to activate the NLRP3 inflammasome, leading to the cleavage and maturation of IL-1β and IL-18 in a caspase-1 dependent manner [36]. Caspase-1 activation and IL-1β production is tied to pyroptosis [37]. In our experiments PFT-induced necroptosis did not require caspase-1. In fact, we observed that infected macrophages did not activate caspase-1, ruling out pyroptosis. We did observe that infected macrophages produced IL-1β, although we did not test if this was cleaved and active IL-1β. Production of IL-1β would be the result of NFκB activation during infection. Its presence in the supernatants would presumably be the result of necroptosis mediated cell lysis.

Kitur et al. showed that inhibition of MLKL decreased IL-1β production during *S*. *aureus* infection of macrophages [7]. As a result, they conclude that necroptosis and the NLRP3 inflammasome share common signaling components. Our studies support this notion, and showed that BMDM deficient in RIP3, NLRP3, ASC, and MyD88 all had pathogen specific reductions in cytotoxicity following infection. Moreover, when macrophage pyroptosis was induced with LPS and nigericin, cytotoxicity could be decreased by pretreatment with necrostatin-5 and a caspase-1 inhibitor (S6 Fig). Our observation that caspase-1 was not involved during PFT-induced necroptosis indicates that the connection between these pathways occurs upstream. The variability we observed between bacteria and their requirement for NLRP3, ASC, and MyD88, suggests that different signals are also in play. Further studies are warranted to dissect the differences and overlap between macrophage pyroptosis and necroptosis during different types of infection.

Necrotic forms of cell death are inflammatory due to the release of alarmins [5]. Our observation that mice depleted of AMs had reduced disease severity during pneumonia, indicates that necroptosis of AMs favors the bacterium. This is not because of AM-mediated clearance of bacteria, since AM depleted mice fared better than controls. As such, possible reasons include nutritional immunity, where dying cells provide sparse nutrients for this bacterial pathogen [38]. Alternatively, that the release of alarmins may somehow alter the microenvironment in a manner that is beneficial to *S*. *marcescens*. Treatment of mice with RIP1 and MLKL inhibitors delayed death of mice challenged intratracheally with a lethal dose of *S*. *marcescens*. N5/C_10_ treatment reduced all measured pathology following sublethal challenge. These improvements were correlated with cytoprotection of lung macrophages and reduced bacterial burden in the lungs. These data agree with our observations with MLKL KO mice that were also protected against macrophage depletion and had less lung damage. Our work expands on that by Kitur et al. who showed inhibition of RIP1 conferred a beneficial effect against *S*. *aureus*. Specifically, we show that blocking of RIP1 or MLKL, but not RIP3 inhibitors, confers a beneficial effect against *S*. *marcescens*. As such, two independent studies now suggest that necroptosis inhibitors may serve as an adjunct therapy for diverse bacterial infections caused by PFT-producing pathogens [7]. This would be an entirely novel approach for treating bacterial infection, one focused on modulation of a host cellular death pathway.

In conclusion, our results demonstrate that PFTs induce necroptosis in macrophages via membrane disruption that results in ion dysregulation, ATP depletion, and the induction of cellular ROS. These alone are sufficient to induce macrophage necroptosis. Our study is the first to suggest that necroptosis is a common form of death following exposure to PFT-producing bacteria. One striking observation is that diverse bacteria capable of producing PFTs all induced RIP1/3-dependent necroptosis. Our findings point to these signaling molecules as viable targets to counteract bacterial infections from significant community and nosocomial pathogens.

## Materials and Methods

### Ethics statement

All mouse experiments were reviewed and approved by the University of Texas Health Science Center at San Antonio Institutional Animal Care and Use Committee under protocol #12030x. Animal care and experimental procedures adhered to Public Law 89–544 (Animal Welfare Act) and its amendments. Public Health Services and the Guide for the Care and Use of Laboratory Animals (U.S. Department of Health & Human Services).

### Bacteria

Clinical isolates of *S*. *marcescens* (strain MB383), *S*. *aureus* (strain NGJ2), and UPEC (strain NGJ3) were obtained from the Division of Infectious Disease, Department of Medicine, at The University of Texas Health Science Center at San Antonio (UTHSCSA). The isogenic unmarked *shlA* deletion mutant in MB383 (strain Δ*shlA*) was constructed via allelic exchange as previously described [18]. *S*. *marcescens*, UPEC, and *S*. *aureus* were grown on Luria-Bertani (LB) agar plates and incubated overnight at 30°C (*S*. *marcescens*) or 37°C (UPEC and *S*. *aureus*). For working cultures, single colonies of these bacteria were transferred to LB broth, incubated at the respective temperature overnight with rolling, and then back-diluted 1:50 for 3h. *S*. *pneumoniae* serotype 4 (strain TIGR4) and its isogenic pneumolysin deficient mutant (Δ*ply*) have been previously described [39]. *S*. *pneumoniae* was grown on tryptic soy blood agar plates overnight and in Todd Hewitt broth at 37°C in 5% CO_2_ for working cultures. Pneumococci at exponential phase of growth, optical density (OD)_620_ = 0.5, were used for the infection of cells. Recombinant pneumolysin, cloned from TIGR4, was purified from transformed *E*. *coli* using published methods [39]. A single colony of *L*. *monocytogenes* (strain 10403S) was grown in fresh made brain-heart infusion broth and grown standing overnight at 30°C. *F*. *tularensis novicida* and *A*. *baumannii* were gifts from Drs. Karl Klose and Bernard Arulanandam (San Antonio, TX) and have been previously described [40, 41]. *A*. *baumannii* was grown in LB at 37°C overnight, then back diluted 1:50 for 3h. *F*. *tularensis novicida* was grown in trypticase soy agar supplemented with 0.1% cysteine at 37°C overnight, then back diluted 1:50 for 3h.

### Infections

Bacteria were grown as described above and all inoculums were prepared by diluting the bacteria with sterile phosphate-buffered saline (PBS) to the final desired concentration. The amount of CFU used for challenge was confirmed after infection by serial dilution of the inoculum, plating on appropriate agar plates, overnight incubation, and extrapolation from colony counts. Forced aspiration of female 6–8 week old BALB/c mice from Charles River Laboratories (Frederick, Maryland) was performed as previously described [18]. We also infected 6 week old RIP3 KO, MLKL KO, and wild type mice in a C57BL/6 background. Briefly, mice were anesthetized with vaporized isoflurane, hung upright by their incisors, and aspiration of the bacteria induced by gently pulling the tongue outward with blunt forceps while pipetting 100 μl of the PBS bacterial suspension into the oropharynx. The tongue was held outward to suppress swallowing until aspiration occurred, typically within 5–10s. For *in vitro* infections, bacteria were diluted in tissue culture media containing 2% fetal bovine serum. Bacteria were added to cells at the designated MOI and duration. In instances where cells required pretreatment, this was for 1h, and then exposed to bacteria or pneumolysin in the presence of the inhibitors. Mice that received pharmacological treatments were injected intraperitoneally with 100 μl of a 100 μM solution.

### Cells

Cell lines used were MH-S and THP-1 [42, 43]. All-trans retinoic acid (1 μM) was added to the media for 3 days to differentiate THP-1 monocytes to macrophage-like cells. Bone marrow was harvested from the femurs and tibias of mice and BMDM were derived as previously described [44]. Alveolar macrophages were obtained from BALF of euthanized mice. Cells were plated at a final concentration of 5 x 10^4^ in 96-well plates and were incubated at 37°C, 5% CO_2_. Unless stated otherwise, macrophage death was evaluated by detection of the cytoplasmic enzyme LDH using the Cytotox 96 Assay kit (Promega, Madison, WI).

### Microscopy

Frozen lung tissue sections and cells grown on slides were used for microscopy. Histology sections were stained with hematoxylin and eosin. For fluorescent microscopy, primary rat monoclonal antibody (CIone: A3-1, Abcam, Cambridge, MA) to F4/80 or rabbit polyclonal to MLKL (Abcam) was diluted at 1:50 in PBS/3% goat serum/3% BSA and incubated over the tissue sections for 1h and then washed with PBS. Goat anti-rat antibody conjugated to FITC or goat anti-rabbit antibody conjugated to FITC (Jackson Immuno Research, West Grove, PA) was used as secondary antibody, diluted at 1:1,000, and incubated over sections for 1h. Membrane staining was done using a Vybrant Lipid Raft Labeling kit (Molecular Probes, Eugene, OR) following manufacturer instruction. Images were captured using a Zeiss AxioXam MRm Rev3 and/or MRc cameras attached to a Zeiss AxioImager Z1 epifluorescent microscope (Carl Zeiss, Thornwood, NY). Mean pixels per frame were calculated using ImageJ64 software.

### Western blot

Whole cell extracts were prepared with RIPA buffer (150mM NaCl, 1% NP-40, 0.5% DOC, 0.1% SDS and 50mM Tris) containing protease inhibitors (Sigma). Biological sample in SDS sample buffer were separated by SDS-PAGE and proteins transferred onto nitrocellulose using a semi-dry electrophoretic system (Bio-Rad, Hercules, CA). Western blot was performed using standard methods with antibody against MLKL, pMLKL, RIP3 (Abcam) at 1:1,000 dilutions. Secondary antibody, horseradish peroxidase conjugated goat anti-rabbit, was used at 1:10,000 dilution (Jackson ImmunoResearch, West Grove, PA). To confirm protein load, membranes were stripped and re-probed with antibody against actin at 1:10,000 (Bethyl Laboratories, Montgomery, TX).

### ATP, ROS, and Cytochrome C measurement

ATP concentration in cells was measured using Cell Titer-Glo, Luminescent Viability Assay (Promega, Madison, WI). Briefly, CellTiter-Glo reagent was added to each well (100 μl) and cellular contents were mix for 2–3m on an orbital shaker. Afterwards, the plate was incubated for 10m to stabilize luminescent signal and luminescence was read in a BioTek Synergy H4 plate reader (BioTek, Winooski, VT). Controls wells without cells and untreated cells were used to obtain background and maximal luminescence. ROS concentration in cells was measured using the cell-permeant 2', 7’-dichlorodihydrofluorescein diacetate indicator of ROS (H2-DCF), (Life Technologies, Grand Island, NY). ROS probe was added to each well to a final working concentration of ~10 mM, followed by a 1h incubation at 37°C. After incubation, a short recovery time of 20m was allowed for cellular esterases to hydrolyze the acetoxymethyl ester or acetate groups. Fluorescence was measured with a BioTek Synergy H4 plate reader. Cytochrome C levels were measured by enzyme-linked immunosorbent assay (ELISA) (Abcam, Cambridge, England) following the manufacturer’s instructions.

### Depletion of Alveolar macrophages

Lung macrophages were depleted using clodronate-containing liposomes [7]. Clodronate and PBS containing liposomes were obtained from ClodronateLiposomes.org (Amsterdam, Netherlands). Mice were administered liposomes intratracheally 24h before infection.

### Flow cytometry

For F4/80 positive cells staining, 50 μl of BALF was transferred to staining vessel, then blocked with FcBlock (0.25 μl per 50 μl of cell suspension), incubated 15m on ice. Primary antibody was added (0.5–1 μl of antibody per 50 μl of staining buffer) and incubated 30m on ice in dark. Then cells were pelleted and washed twice in FACS buffer. Finally cells were re-suspended in 50 μl fix buffer (2% PFA in PBS) and incubated for 30m then spun, washed twice in PBS and re-suspended in 300 μl PBS. For alveolar macrophage counts, cells were incubated with Fc-Block after erythrocyte lysis, and stained with fluorochrome-conjugated MAbs including CD45-BV605 (clone 30-F11), CD4-PE-Cy7 (cloneRM4-4), CD8-PE-Cy7 (clone53-637), CD19-PE-Cy7 (clone 6D5), Ly6G-FITC (clone 1A8), Ly6C-PE (clone HK1.4), F4/80-APC (clone BM8), MHC II-PerCP-Cy5.5 (clone M5/114.15.2), CD11b-APC-eFluor 780 (clone M1/710), CD11c –eFluor450 (clone N418) purchased from BioLegend or eBioscience. Flow cytometric studies were performed using LSR II (BD) with subsequent data analysis using FlowJo software (Tree Star). CD4, 8, or 19+ (Lymphocytes), Ly6G+ CD11b^hi^ (Neutrophils), and Ly6C^hi^ CD11b^hi^ (Ly6C^hi^ Monocytes) populations were systematically out gated from CD45+ (leukocytes) population, and then a population with F4/80+ CD11b^low^ CD11c^hi^ MHC II^low^ was gated and defined as alveolar macrophage. For caspase activity fluorescent-labeled inhibitor of caspases (FLICA) was used to stain activation of caspase-1 and 3/7 in MH-S macrophages. According with the manufacturer direction, 2h following cell challenge with *S*. *marcescens* in combination with FLICA, wells were washed with the supernatant set aside. After adding trypsin the rest of the cells were merged with the previously gathered supernatant. Cells were then spun at 220xg for 5m and supernatant discarded. Cells were then spun and re-suspended in apoptosis buffer provided by the manufacturer and read on flow cytometer.

### siRNA

Commercially available siRNA targeting RIP3, MLKL, caspase-1 and caspase-8 (Santa Cruz, Dallas, Texas) was used to transfect THP-1 macrophages following the manufacturer instructions.

### IL-1 β and TNF-α ELISA

IL-1 β and TNF-α were measured in culture supernatants by ELISA following the manufacturer instructions (BD Biosciences, MD, USA).

### Bronchoalveolar lavage Fluid (BALF) and cytospins

BALF was collected and cytospins were performed as previously described [18]. Following tracheostomy, lungs were washed 3 times with 0.5 mL of PBS and collect BALF. BALF was then centrifuged and re-suspended in 0.5 mL PBS. Cell concentration was determined using the Cellometer Slide Chamber (Nexcelom Biosciences. Slides were stained for differentiation of blood cell types with Protocol Hema 3 stain set (Fisher Scientific).

### Inhibitors and other chemicals

Necrostatin-1, -5, -7, Glycine, Coenzyme Q_10_, Resveratrol, DPPC, DPI, NAC, rotenone, antimycin, and TTFA were obtained from Sigma (Aldrich, St. Louis, MO). Necrostatin-1s and GSK’872 were obtained from BioVision (Milpitas, California). Necrosulfonamide was obtained from Tocris Bioscience (QL, United Kingdom). Caspase inhibitors: Z-VAD-FMK, general caspase inhibitor; Z-WEHD-FMK, caspase-1 inhibitor; Z-VDVAD-FMK, caspase-2 inhibitor; Z-DEVD-FMK, caspase-3 inhibitor; Z-YVAD-FMK, caspase-4 inhibitor; Z-VEID-FMK, caspase-6 inhibitor; Z-IETD-FMK, caspase-8 inhibitor; Z-LEHD-FMK, caspase-9 inhibitor; Z-AEVD-FMK and caspase-10 inhibitor were obtained from R&D Systems (Minneapolis, MN). GW806742X, MLKL inhibitor was obtained from SYNkinase (Australia). All chemical were used at the stated concentration in the figure legends.

### Statistical analysis

Prism 5 (GraphPad Software, La Jolla CA) was used for graph development and statistical analysis. Survival curves were made using the Kaplan Meier method and significance calculated using the log-rank test. Student’s *t*-test and Mann-Whitney tests were applied for two-group comparisons as indicated, and nonparametric ANOVA (Kruskal-Wallis) and Dunn's *post hoc* analyses were used for multiple-group comparisons.

## Supporting Information

S1 Fig Knockdown of RIP3 and MLKL with siRNA. Western blot for **A)** RIP3 and **B)** MLKL levels in THP-1 macrophages transfected with siRNA targeting RIP3 and MLKL, respectively, with a scrambled siRNA control. **C)** Percent transfection efficacy from THP-1 macrophages transfected with FITC-labeled control siRNA.
(TIFF)

S2 Fig MLKL activation is PFT dependent. **A)** Immunofluorescence for MLKL (Green) in MH-S cells following their challenge with wild type *S*. *marcescens* (*Sma*), Δ*shlA*, *S*. *pneumoniae* (*Spn*), Δ*ply*, recombinant pneumolysin (rPly), and heat inactivated rPly (HI-rPly). In designated instances cells were pre-treated with necrostatin-5 (Nec-5; 100 μM). Nuclei were stained with DAPI. **B)** TNFα/ZVAD induced necroptosis (6h) incites localization of MLKL to the plasma membrane in a similar manner as panel A. **C)** Immunofluorescence staining for MLKL (green) in MH-S cells treated with pneumolysin (rPly) to induce necroptosis, with and without pretreatment with necrosulfonamide (NSA) (100μM). Images are representative of 3 experiments.
(TIFF)

S3 Fig Ion dysregulation can induce MLKL aggregation. Immunofluorescence staining for MLKL (green) in MH-S cells treated with ionomycin (io), nigericin (ni), and gramicidin (gr) with and without pretreatment with necrostatin-5 (N5). Nuclei stained with DAPI (blue). Images are representative of 3 experiments.
(TIFF)

S4 Fig CoQ10 and not soluble ATP can decrease MLKL aggregation. Immunofluorescence staining for MLKL (green) in MH-S cells treated with recombinant pneumolysin (rPly) to induce necroptosis, with and without pretreatment with ATP or CoQ_10_. Nuclei stained with DAPI (blue). Images are representative of 3 experiments.
(TIFF)

S5 Fig Blocking of necroptosis signaling and mitochondrial support decreases pathology associated with *S*. *marcescens* pneumonia. **A)** Representative hematoxylin and eosin stained lung sections and their corresponding pathology score. Note mice that received N5/C_10_ showed less destruction of perivascular tissue, cell metaplasia, edema and hemorrhage than PBS controls (n = 3/cohort). **B**) Representative stained lung sections from an MLKL KO mouse infected with *S*. *marcescens* versus wild type control (n = 3/cohort) (20X). **C)** Images of BALF taken from *S*. *marcescens* infected mice (low dose) following pretreatment with PBS or N5/CoQ_10_.
(TIFF)

S6 Fig Nigericin induced pyroptosis can be blocked by RIP1 inhibition. MH-S cells were treated with lipopolysaccharide and nigericin to induced pyroptosis. Cells were pretreated with necrostatin-5 (n5; 100 μM), ZVAD (10 μM), Caspase-1 inhibitor (C1; 10 μM). Dunn’s multiple-comparison post-test was used: *, *P* ≤ 0.05. Data are representative of ≥3 separate experiments, each with 8 biological replicates.
(TIFF)
