# Supplementary figures and images for: Pore-Forming Toxins Induce Macrophage Necroptosis during Acute Bacterial Pneumonia
Source: PLoS Pathog. 2015 Dec 11;11(12):e1005337. doi: 10.1371/journal.ppat.1005337 (PMC4676650; doi:10.1371/journal.ppat.1005337)

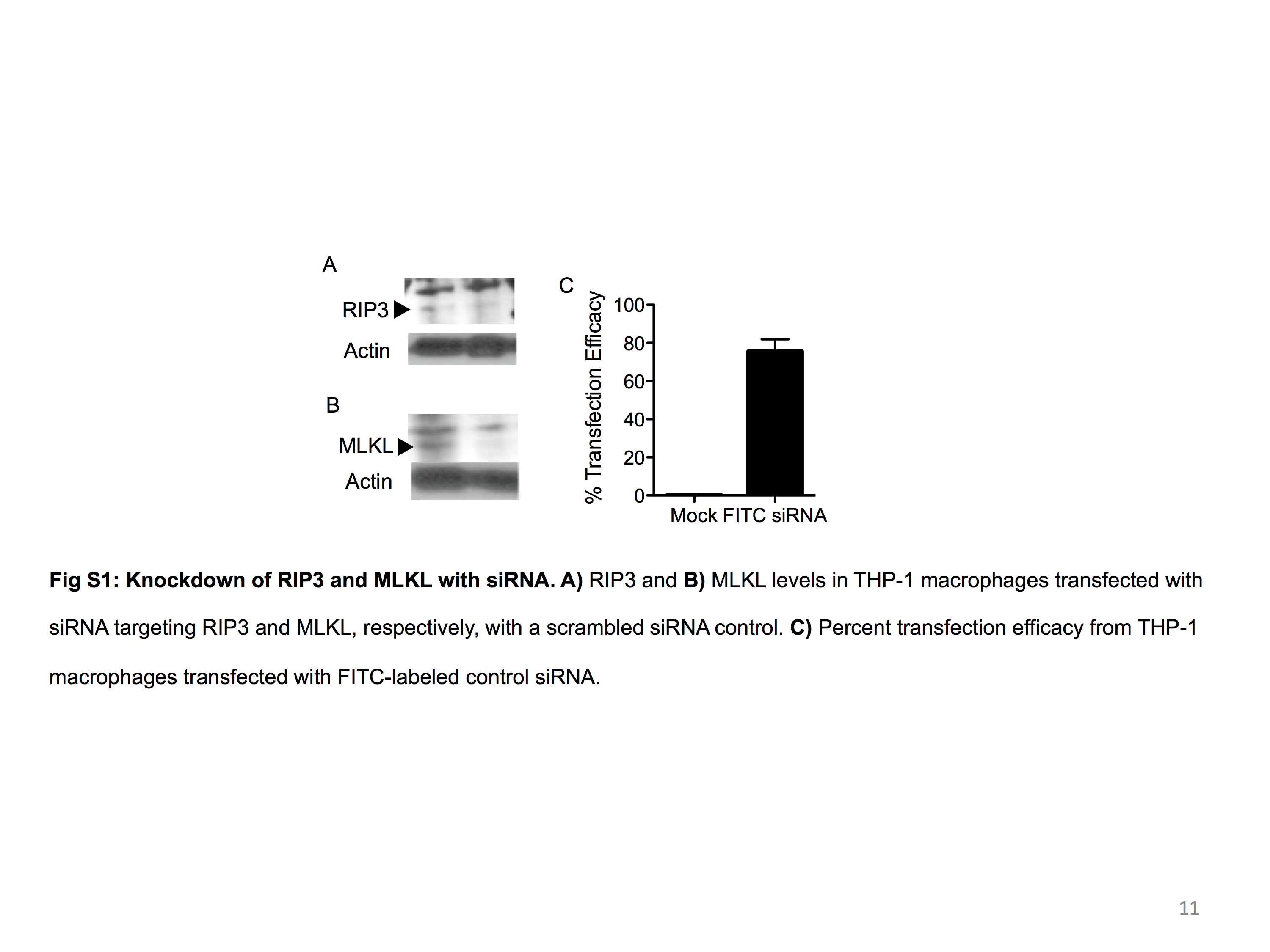

Supplement: S1 Fig — Western blot for A) RIP3 and B) MLKL levels in THP-1 macrophages transfected with siRNA targeting RIP3 and MLKL, respectively, with a scrambled siRNA control. C) Percent transfection efficacy from THP-1 macrophages transfected with FITC-labeled control siRNA. (TIFF) [file ppat.1005337.s001.tiff]

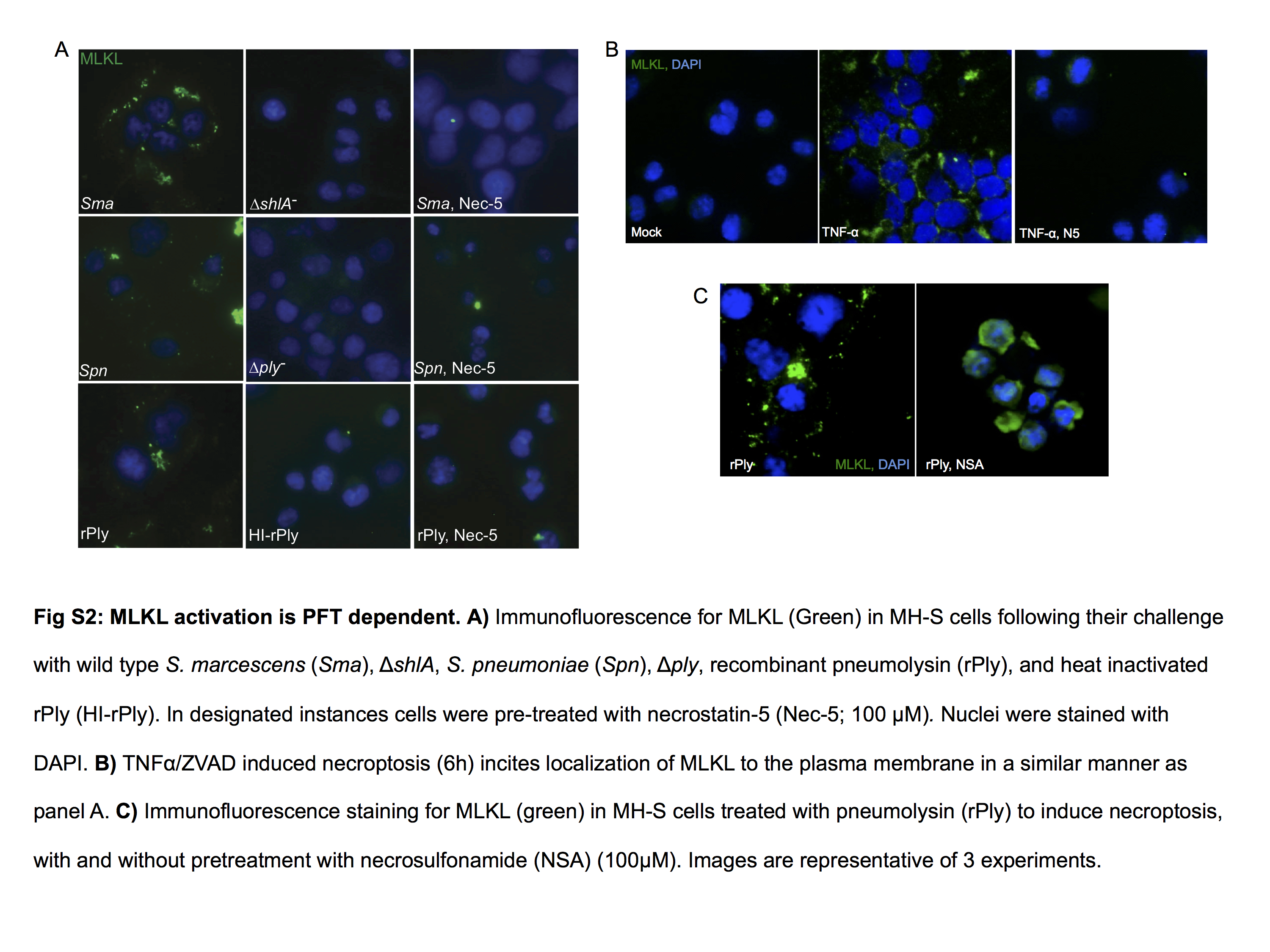

Supplement: S2 Fig — A) Immunofluorescence for MLKL (Green) in MH-S cells following their challenge with wild type S. marcescens (Sma), ΔshlA, S. pneumoniae (Spn), Δply, recombinant pneumolysin (rPly), and heat inactivated rPly (HI-rPly). In designated instances cells were pre-treated with necrostatin-5 (Nec-5; 100 μM). Nuclei were stained with DAPI. B) TNFα/ZVAD induced necroptosis (6h) incites localization of MLKL to the plasma membrane in a similar manner as panel A. C) Immunofluorescence staining for MLKL (green) in MH-S cells treated with pneumolysin (rPly) to induce necroptosis, with and without pretreatment with necrosulfonamide (NSA) (100μM). Images are representative of 3 experiments. (TIFF) [file ppat.1005337.s002.tiff]

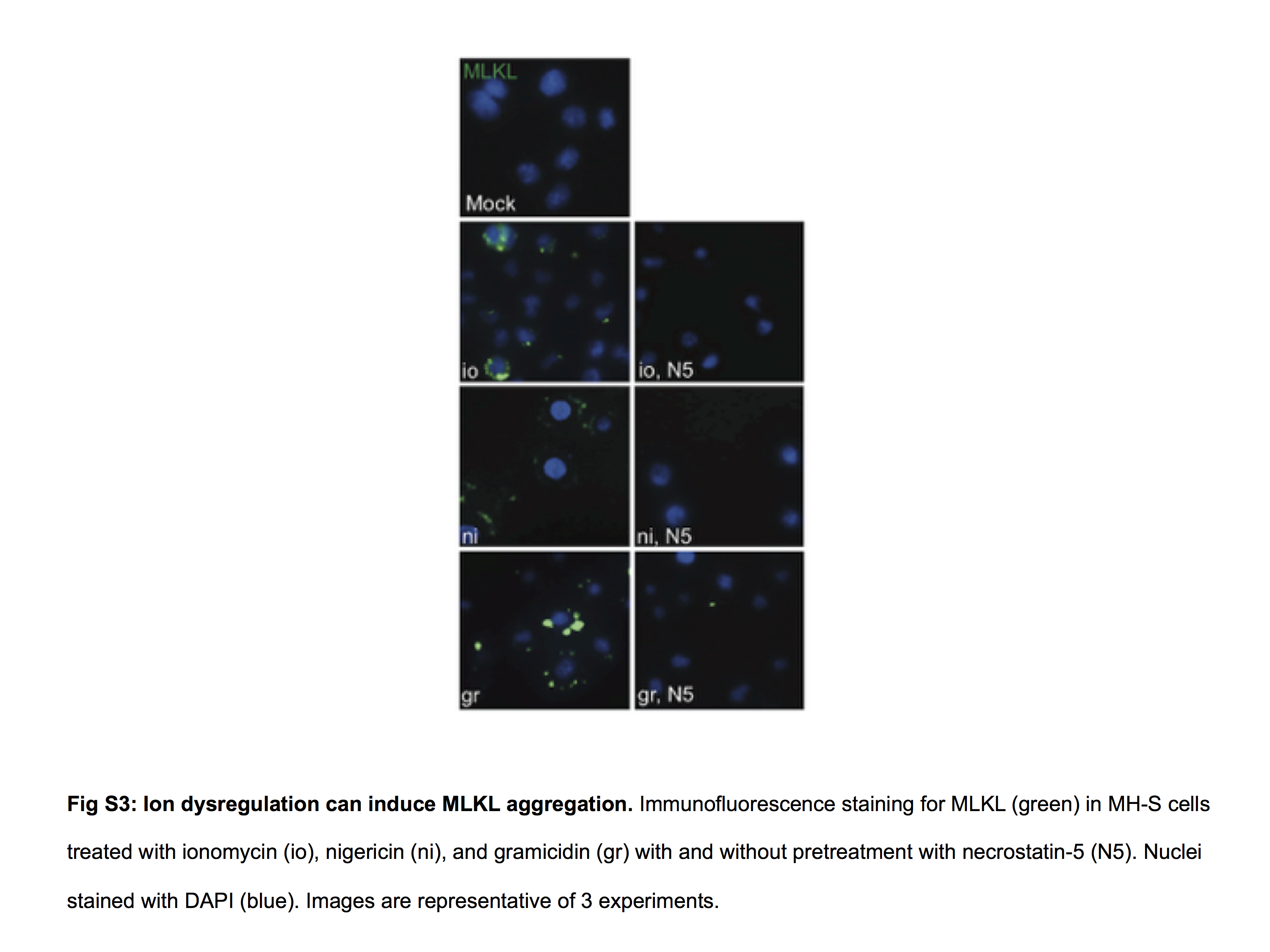

Supplement: S3 Fig — Immunofluorescence staining for MLKL (green) in MH-S cells treated with ionomycin (io), nigericin (ni), and gramicidin (gr) with and without pretreatment with necrostatin-5 (N5). Nuclei stained with DAPI (blue). Images are representative of 3 experiments. (TIFF) [file ppat.1005337.s003.tiff]

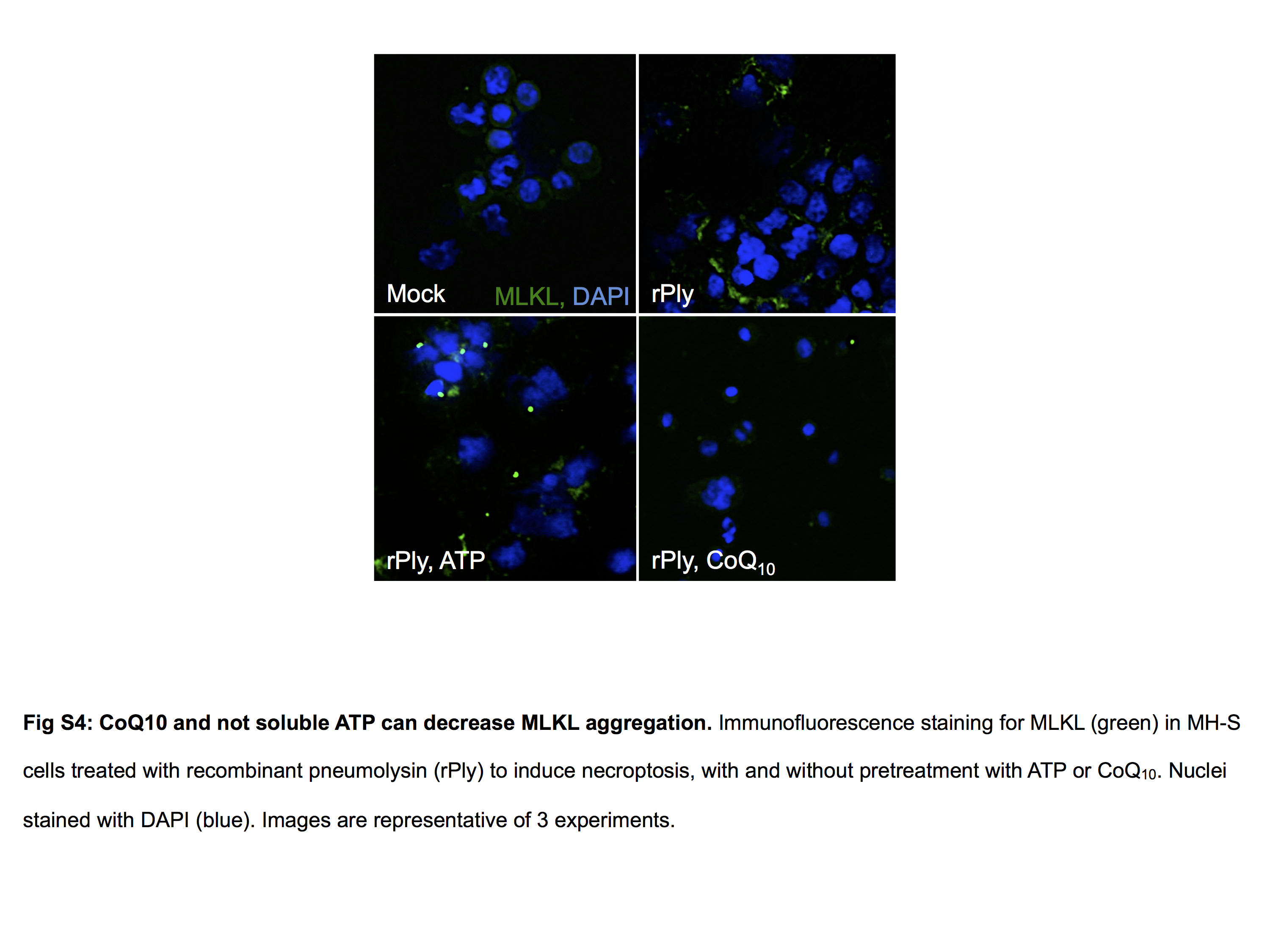

Supplement: S4 Fig — Immunofluorescence staining for MLKL (green) in MH-S cells treated with recombinant pneumolysin (rPly) to induce necroptosis, with and without pretreatment with ATP or CoQ10. Nuclei stained with DAPI (blue). Images are representative of 3 experiments. (TIFF) [file ppat.1005337.s004.tiff]

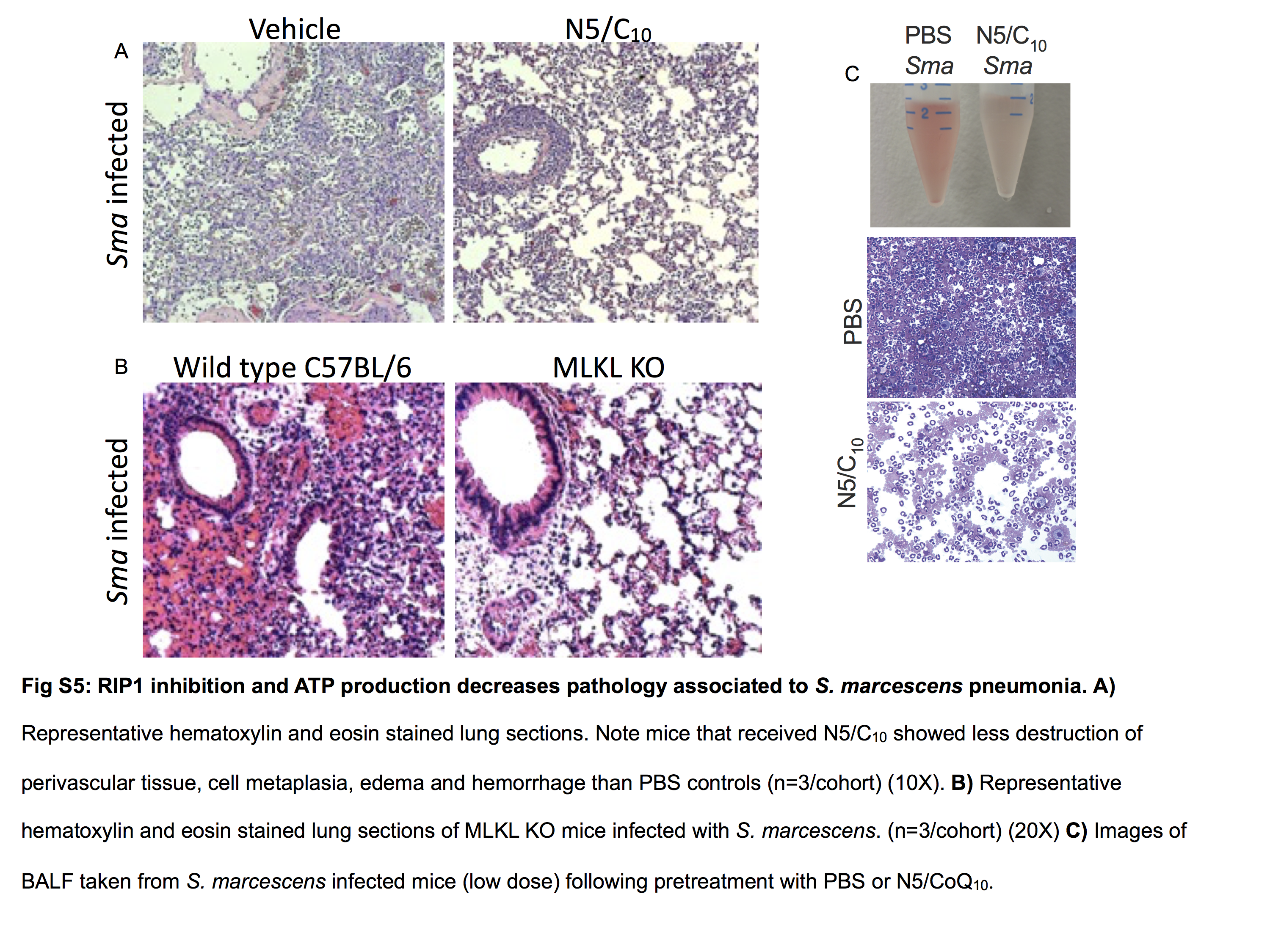

Supplement: S5 Fig — A) Representative hematoxylin and eosin stained lung sections and their corresponding pathology score. Note mice that received N5/C10 showed less destruction of perivascular tissue, cell metaplasia, edema and hemorrhage than PBS controls (n = 3/cohort). B) Representative stained lung sections from an MLKL KO mouse infected with S. marcescens versus wild type control (n = 3/cohort) (20X). C) Images of BALF taken from S. marcescens infected mice (low dose) following pretreatment with PBS or N5/CoQ10. (TIFF) [file ppat.1005337.s005.tiff]

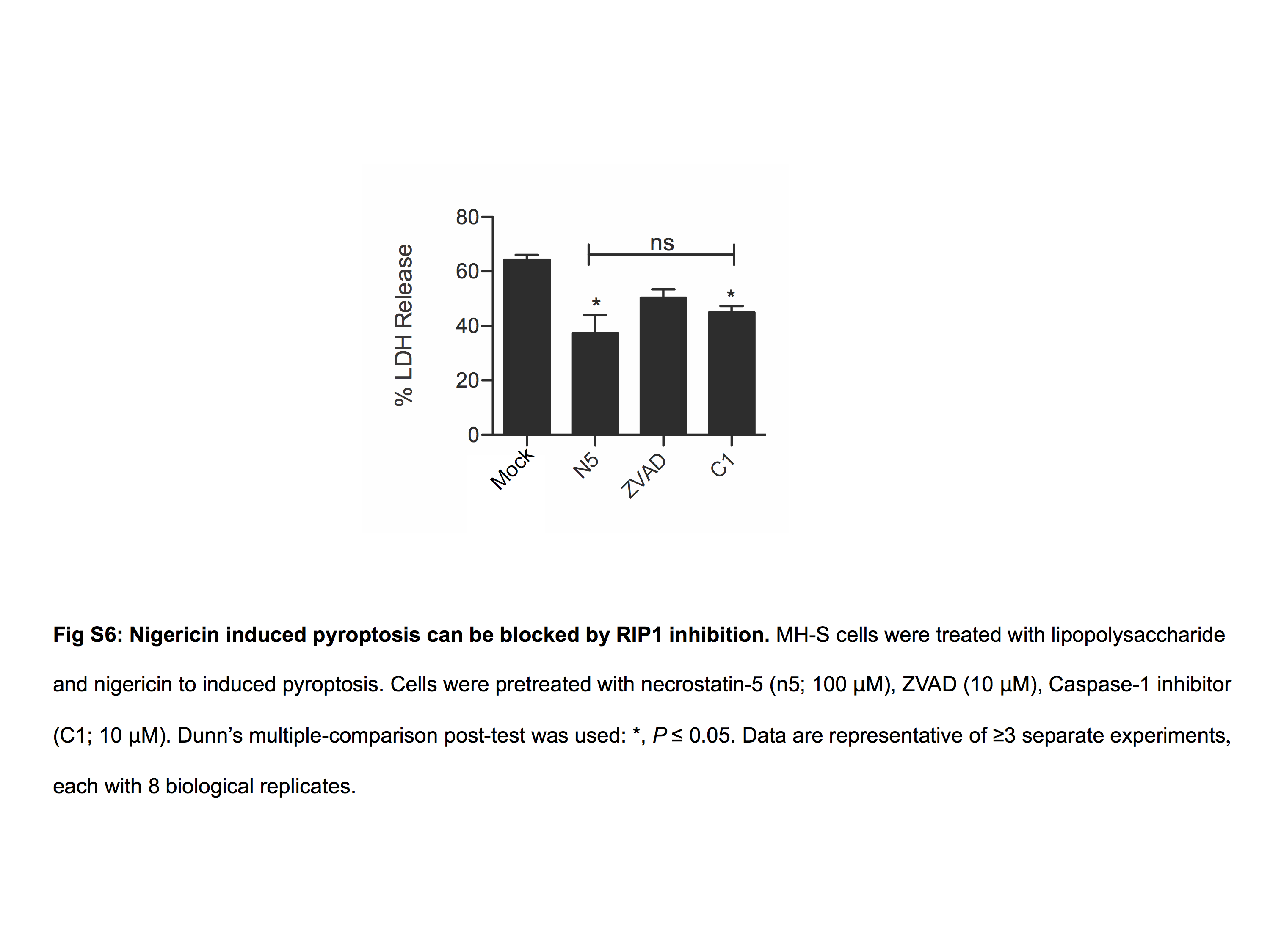

Supplement: S6 Fig — MH-S cells were treated with lipopolysaccharide and nigericin to induced pyroptosis. Cells were pretreated with necrostatin-5 (n5; 100 μM), ZVAD (10 μM), Caspase-1 inhibitor (C1; 10 μM). Dunn’s multiple-comparison post-test was used: *, P ≤ 0.05. Data are representative of ≥3 separate experiments, each with 8 biological replicates. (TIFF) [file ppat.1005337.s006.tiff]
